# Identification of BiP as a CB_1_ Receptor-Interacting Protein That Fine-Tunes Cannabinoid Signaling in the Mouse Brain

**DOI:** 10.1523/JNEUROSCI.0821-21.2021

**Published:** 2021-09-22

**Authors:** Carlos Costas-Insua, Estefanía Moreno, Irene B. Maroto, Andrea Ruiz-Calvo, Raquel Bajo-Grañeras, David Martín-Gutiérrez, Rebeca Diez-Alarcia, M. Teresa Vilaró, Roser Cortés, Nuria García-Font, Ricardo Martín, Marc Espina, Joaquín Botta, Silvia Ginés, Peter J. McCormick, José Sánchez-Prieto, Ismael Galve-Roperh, Guadalupe Mengod, Leyre Urigüen, Giovanni Marsicano, Luigi Bellocchio, Enric I. Canela, Vicent Casadó, Ignacio Rodríguez-Crespo, Manuel Guzmán

**Affiliations:** ^1^Centro de Investigación Biomédica en Red sobre Enfermedades Neurodegenerativas, Madrid, 28031, Spain; ^2^Department of Biochemistry and Molecular Biology, Instituto Universitario de Investigación Neuroquímica, Complutense University, Madrid, 28040, Spain; ^3^Instituto Ramón y Cajal de Investigación Sanitaria, Madrid, 28034, Spain; ^4^Department of Biochemistry and Molecular Biomedicine, Faculty of Biology, Institute of Biomedicine of the University of Barcelona, University of Barcelona, Barcelona, 08028, Spain; ^5^Department of Pharmacology, University of the Basque Country/Euskal Herriko Unibertsitatea, Leioa, 48940, Spain; ^6^Centro de Investigación Biomédica en Red de Salud Mental, Madrid, 28029, Spain; ^7^Institut d'Investigacions Biomèdiques August Pi i Sunyer, Barcelona, 08036, Spain; ^8^Department of Neurosciences and Experimental Therapeutics, Institut d' Investigacions Biomèdiques de Barcelona, Consejo Superior de Investigaciones Científicas, Barcelona (IIBB-CSIC), 08036, Spain; ^9^Instituto de Investigación, Sanitaria del Hospital Clínico San Carlos, Madrid, 28040, Spain; ^10^Department of Biomedicine, School of Medicine, Institute of Neuroscience, University of Barcelona, Barcelona, 08036, Spain; ^11^Centre for Endocrinology, William Harvey Research Institute – Barts, and London School of Medicine and Dentistry, Queen Mary University of London, EC1M 6BQ, London, United Kingdom; ^12^Institut National de la Santé et de la Recherche Médicale University of Bordeaux, NeuroCentre Magendie, Physiopathologie de la Plasticité Neuronale, Bordeaux, 33077, France

**Keywords:** BiP, cannabinoid, cell signaling, G-protein-coupled receptor, neurotransmission, protein–protein interaction

## Abstract

Cannabinoids, the bioactive constituents of cannabis, exert a wide array of effects on the brain by engaging Type 1 cannabinoid receptor (CB_1_R). Accruing evidence supports that cannabinoid action relies on context-dependent factors, such as the biological characteristics of the target cell, suggesting that cell population-intrinsic molecular cues modulate CB_1_R-dependent signaling. Here, by using a yeast two-hybrid-based high-throughput screening, we identified BiP as a potential CB_1_R-interacting protein. We next found that CB_1_R and BiP interact specifically *in vitro*, and mapped the interaction site within the CB_1_R *C*-terminal (intracellular) domain and the BiP *C*-terminal (substrate-binding) domain-α. BiP selectively shaped agonist-evoked CB_1_R signaling by blocking an “alternative” G_q/11_ protein-dependent signaling module while leaving the “classical” G_i/o_ protein-dependent inhibition of the cAMP pathway unaffected. *In situ* proximity ligation assays conducted on brain samples from various genetic mouse models of conditional loss or gain of CB_1_R expression allowed to map CB_1_R-BiP complexes selectively on terminals of GABAergic neurons. Behavioral studies using cannabinoid-treated male BiP^+/−^ mice supported that CB_1_R-BiP complexes modulate cannabinoid-evoked anxiety, one of the most frequent undesired effects of cannabis. Together, by identifying BiP as a CB_1_R-interacting protein that controls receptor function in a signaling pathway- and neuron population-selective manner, our findings may help to understand the striking context-dependent actions of cannabis in the brain.

**SIGNIFICANCE STATEMENT** Cannabis use is increasing worldwide, so innovative studies aimed to understand its complex mechanism of neurobiological action are warranted. Here, we found that cannabinoid CB_1_ receptor (CB_1_R), the primary molecular target of the bioactive constituents of cannabis, interacts specifically with an intracellular protein called BiP. The interaction between CB_1_R and BiP occurs selectively on terminals of GABAergic (inhibitory) neurons, and induces a remarkable shift in the CB_1_R-associated signaling profile. Behavioral studies conducted in mice support that CB_1_R-BiP complexes act as fine-tuners of anxiety, one of the most frequent undesired effects of cannabis use. Our findings open a new conceptual framework to understand the striking context-dependent pharmacological actions of cannabis in the brain.

## Introduction

Preparations of the hemp plant *Cannabis sativa* L. have been used by humankind for millennia. During the last decades, there has been a strong renaissance in the study of the molecular and pharmacological bases of cannabinoid action; and, in concert, many countries have approved the use of cannabinoid-based medicines and standardized preparations of medicinal cannabis (Hill, 2015; Abrams, 2018). Both the therapeutic and the adverse effects of cannabis are mostly attributed to a single molecule, Δ^9^-tetrahydrocannabinol (THC) (Mechoulam et al., 2014). This compound engages and activates two specific G-protein-coupled receptors (GPCRs), designated as cannabinoid CB_1_ receptor (CB_1_R) and cannabinoid CB_2_ receptor (CB_2_R) (Pertwee et al., 2010). CB_1_R is one of the most abundant GPCRs in the mammalian brain (Katona and Freund, 2008; Pertwee et al., 2010; Dudok et al., 2015). It mediates a large number of pharmacological effects of THC, and, on binding endocannabinoids (anandamide and 2-arachidonoylglycerol), participates in the physiological control of multiple processes, such as motor behavior, learning and memory, fear and anxiety, pain, food intake, and energy metabolism (Piomelli, 2003; Mechoulam et al., 2014).

The precise molecular mechanism of CB_1_R action remains unsolved. For example, CB_1_R couples to the inhibitory family of heterotrimeric G-proteins (G_i/o_), but its expression and signaling efficacy differ remarkably between excitatory and inhibitory neurons (Steindel et al., 2013), which could explain, at least in part, the widely reported biphasic effects elicited by THC and other CB_1_R agonists (Bellocchio et al., 2010; Rey et al., 2012; Mechoulam and Parker, 2013). Likewise, under different cellular settings, CB_1_R can signal through other G-protein families, such as G_q/11_ and G_s_ (Lauckner et al., 2005; Priestley et al., 2017). Furthermore, CB_1_R activation protects neurons from death in a wide array of pathologic conditions (Fernández-Ruiz, 2019), while it triggers apoptosis of brain cancer cells (Velasco et al., 2012). How these striking differences in signaling efficacy, G-protein coupling, and biological response occur is not understood. Together, these observations suggest a cell population-selective action of CB_1_R colligated to the physiopathological context of the target cell expressing the receptor. Understanding how CB_1_R evokes such varying responses is important to clarify the neurobiological role of the endocannabinoid system and, potentially, to improve the design of CB_1_R-targeted therapies.

Interaction with regulatory proteins represents one of the pivotal molecular processes by which GPCR-evoked signaling is affected. Diverse subsets of these interacting proteins assist GPCRs during biosynthesis, trafficking, activation, desensitization, and degradation (Maurice et al., 2011). Aside from the most ubiquitous GPCR-associated proteins (i.e., G-proteins, β-arrestins, and GPCR kinases), specific interacting partners have been identified for particular types of receptors (e.g., NHERF proteins for adrenergic receptors and others, and Homer proteins for metabotropic glutamate receptors and others) (Wheeler et al., 2007; Magalhaes et al., 2012). Regarding CB_1_R, several intracellular proteins (led by CRIP1a) (Howlett et al., 2010; Guggenhuber et al., 2016), as well as membrane-anchored GPCRs (e.g., serotonin 5-HT_2A_ and adenosine A_2A_ receptors) (Viñals et al., 2015; Moreno et al., 2018), have been proposed as receptor interactors. However, most studies on these putative protein complexes have been conducted *in vitro*, and only subtle effects have been unraveled so far *in vivo*.

Here, we hypothesized that unidentified neuron population-specific CB_1_R-interacting proteins modulate cannabinoid signaling in the brain. By using a yeast two-hybrid (Y2H)-based approach, complemented with a wide array of molecular, genetic, pharmacological, and behavioral procedures, we identified the protein BiP as a new modulator of biased signaling of CB_1_R, and defined the molecular features, signal-transduction consequences, neuroanatomical mapping, and behavioral outcomes of the CB_1_R-BiP interaction.

## Materials and Methods

### 

#### 

##### Gene constructs

Y2H vectors were generated by PCR and subsequent restriction cloning by using pGBT9 and pGAD as vectors (ClonTech, TaKaraBio). Short amino-acid stretches (CB_1_R mutants) were ligated by using long annealing oligonucleotides with protruding overhangs. The cDNA encoding full-length BiP was provided by Valerie Petegnief (Institute for Biomedical Research of Barcelona), and expression vectors encoding nontagged (pcDNA3.1+ backbone; Thermo Fisher Scientific), GFP-tagged (pEGFP-C2 backbone; ClonTech), and recombinant bacterial-expression [pBH4 backbone (Merino-Gracia et al., 2016b)] versions were built as well by PCR and restriction cloning. BiP-ΔIR comprised BiP amino acids 1-308. 3XFLAG-tagged versions were obtained by using IVA cloning (García-Nafría et al., 2016) with pcDNA3.1+ plasmids as templates. pcDNA3.1-HA-CB_1_R, pcDNA3.1-CB_1_R-myc, CB_1_R-Rluc, CB_1_R-GFP, and pcDNA3.1-A1R constructs had been generated previously in our laboratory. Single phosphomimetic mutants of CB_1_R–carboxyl-terminal domain (CTD), as well as the CB_1_R-S452D-Rluc construct, were obtained through QuickChange mutagenesis with the aforementioned plasmids as templates. pcDNA3.1-CB_2_R was provided by Cristina Sánchez (Complutense University of Madrid) and used to construct the corresponding Y2H vector. pCEFL-GFP and pCEFL-GFP-GRK2 plasmids were given by J. Silvio Gutkind (University of California San Diego). All constructs were validated by Sanger sequencing before use.

##### Y2H

Screening of the library was performed following the manufacturer's instructions (MatchMaker system, TaKaraBio). Plasmids of positive transformants were isolated and subsequently sequenced by standard procedures. Directed Y2H experiments were conducted as previously reported (Merino-Gracia et al., 2016a). Yeasts were transformed with plasmids containing the GAL4 binding domain and the GAL4 activation domain following a lithium acetate-based method. Double transformants were placed on Leu/Trp/His-deficient plates in the presence of 12 mm 3-aminotriazole (triple dropout plates) as well as only Leu/Trp-deficient plates. Interacting proteins expressed within the same yeast allowed colonies that could rescue growth in triple-dropout plates and were capable to hydrolyze X-Gal.

##### Protein expression and purification

pBH4 plasmids encoding His6-tagged BiP, BiP-IR (amino acids 497-654), or CB_1_R-CTD (amino acids 400-472) were used to transform competent BL21 DE3 *Escherichia coli*. Typically, 2 L of bacterial culture in 2xYT (16 g/L tryptone, 10 g/L yeast extract, 5 g/L NaCl, pH 7.0) was used for recombinant protein expression. Protein expression was induced by addition of 0.5 mm isopropyl 1-thio-β-D-galactopyranoside (Panreac Química SAU) and incubation overnight at 30°C with 250 rpm aeration rate. Bacterial cells were pelleted and frozen at −20°C until used for protein purification.

Bacterial cell lysis was conducted in ice-cold lysis buffer (100 mm Tris-HCl, 100 mm NaCl, 10 mm imidazole, pH 7.0) with continuous shaking in the presence of protease inhibitors (1 μg/ml aprotinin, 1 μg/ml leupeptin, 200 μm PMSF), 0.2 g/L lysozyme, and 5 mm β-mercaptoethanol, followed by four cycles of sonication on ice. The cell lysate was clarified by centrifugation at 10,000 × *g* and filtration through porous paper. Recombinant His6-tagged proteins were sequentially purified on a nickel-nitrilotriacetic acid affinity column. After extensive washing (50 mm Tris, 100 mm NaCl, 25 mm imidazole, pH 7.0), proteins were eluted with elution buffer (50 mm Tris, 100 mm NaCl, 250 mm imidazole, pH 7.0; supplemented with protease inhibitors). Protein purity was confirmed by SDS-PAGE and Coomassie Brilliant Blue or Silver staining. Pure protein solutions were concentrated by centrifugation in Centricon tubes (Millipore).

##### Fluorescence polarization

His6-tagged CB_1_R-CTD (amino acids 400-472) was labeled with 5-(iodoacetamido)fluorescein (5-IAF) by standard procedures. Briefly, the FITC dye was dissolved in DMSO, and the labeling reaction was performed in sodium bicarbonate buffer, pH 9.0, with a threefold molar excess of dye for 1 h at 25°C, protected from light. Subsequently, a 1.00 Da cutoff dialysis membrane was used to eliminate nonreacted 5-IAF compound. After extensive dialysis, the concentration of the labeled peptide was calculated using the value 68,000 cm^−1^ m^−1^ as the molar extinction coefficient of the dye at pH 8.0 at 494 nm. Saturation binding experiments were performed essentially as described previously (Merino-Gracia et al., 2016a) with a constant concentration of 100 nm 5-IAF-CB_1_R-CTD. The fluorescence polarization values obtained were fitted to the equation (FP – FP_0_) = (FP_Max_ – FP_0_)[BiP or BiP-IR]/(*K*_d_ + [BiP or BiP-IR]), where FP is the measured fluorescence polarization, FP_Max_ is the maximal fluorescence polarization value, FP_0_ is the fluorescence polarization in the absence of added BiP or BiP-IR, and *K*_d_ is the dissociation constant as determined with GraphPad Prism version 8.0.1 (GraphPad Software). FP was expressed as milli-FP units (mFP; net FP × 1000). Each representative curve shown is the mean of three internal replicates.

##### Cell culture, transfection, and incubation

The HEK-293T cell line was obtained from the American type Culture Collection. Cells were grown in DMEM supplemented with 10% FBS (Thermo Fisher Scientific), 1% penicillin/streptomycin, 1 mm Na-pyruvate, 1 mm L-glutamine, and essential medium nonessential amino acids solution (diluted 1/100) (all from Invitrogen). Cells were maintained at 37°C in an atmosphere with 5% CO_2_ in the presence of the selection antibiotic (zeocin at 0.22 mg/ml, Thermo Fisher Scientific), and were periodically checked for the absence of mycoplasma contamination. Cell transfections were conducted with polyethyleneimine (Polysciences) in a 4:1 mass ratio to DNA according to the manufacturer's instructions. Double transfections were performed with equal amounts of the two plasmids. In all cases, 48 h after transfection, cells were washed twice in quick succession, detached, and harvested for further procedures. To control cell number, protein concentration in the samples was determined with a Bradford assay kit (Bio-Rad).

Drug treatments to assess CB_1_R-evoked signaling were conducted as follows. A 10-cm-diameter plate of transfected cells was trypsinized and seeded on a 6-well plate at a density of 0.75 × 10^6^ cells per well. Six hours later, cells were serum-starved overnight. Then, WIN-55212-2 (Tocris Bioscience; 100 nm final concentration) or vehicle (DMSO, 0.1% v/v final concentration) was added for 5, 10, or 15 min. Gα_q/11_ inhibition was achieved by adding YM-254890 (Focus Biomolecules; 1 μm final concentration) or vehicle (DMSO, 0.1% v/v final concentration) 30 min before WIN-55212-2 (100 nm final concentration) or vehicle (DMSO, 0.1% v/v final concentration). All incubations were conducted in triplicate. Cells were subsequently washed with ice-cold PBS, snap-frozen in liquid nitrogen, and harvested at −80°C for Western blot analyses.

##### *In situ* proximity ligation assay (PLA)

BiP-CB_1_R complexes were detected by using the Duolink In Situ PLA Detection Kit (Sigma-Aldrich) following the manufacturer's instructions. Synaptosomal preparations were incubated with a rabbit-anti-CB_1_R antibody (1:500, Frontier-Institute, #CB1-Rb-Af530) and a mouse anti-GRP78/BiP antibody (1:500, Santa Cruz Biotechnology, #sc-376768). Negative controls were performed with just one primary antibody. Ligations and amplifications were performed with In Situ Detection Reagent Red (Sigma-Aldrich), and coverslips were mounted in DAPI-containing mounting medium. Samples were analyzed with a Leica SP2/SP8 confocal microscope (Leica Microsystems). For each FOV, a stack of two channels (one per staining) and 9-13 *Z* stacks with a step size of 0.3 µm were acquired with a 63× oil-immersion objective and processed with ImageJ software (National Institutes of Health). Representative images for each condition were prepared for figure presentation by applying color adjustments uniformly with Adobe Photoshop version CS6.

For PLA imaging in brain sections, mice were deeply anesthetized and immediately perfused transcardially with PBS followed by 4% PFA/PB. Brains were removed and postfixed overnight in the same solution, cryoprotected by immersion in 10%, 20%, 30% gradient sucrose (24 h for each sucrose gradient) at 4°C, and then frozen in dry ice-cooled methylbutane. Serial coronal or sagittal cryostat sections (30-μm-thick) through the whole brain were collected in cryoprotective solution and stored at −20°C until PLA experiments were performed. Immediately before the assay, mouse brain sections were mounted on glass slides, washed in PBS, permeabilized with PBS containing 0.01% Triton X-100 for 10 min, and successively washed with PBS. Interactions were detected with Duolink In Situ PLA Detection and In Situ Detection Reagent Red Kits. A mixture of the primary antibodies [mouse anti-GRP78/BiP antibody (1:100, Santa Cruz Biotechnology, #sc-376768) and rabbit anti-CB_1_R antibody (1:100, Thermo Fisher Scientific, #PA1-745)] was used. Samples were analyzed in a Leica SP2 confocal microscope (Leica Microsystems) equipped with an apochromatic 63× oil-immersion objective (1.4 numerical aperture), and a 405 nm and a 561 nm laser lines. For each FOV, a stack of two channels (one per staining) and 9-13 *Z* stacks with a step size of 1 µm were acquired. Images were opened and processed with ImageJ software (National Institutes of Health). Quantification of cells containing one or more red dots versus total cells (blue nuclei) was determined by using the Fiji package (https://imagej.net/software/fiji/). Nuclei and red dots were counted on the maximum projections of each image stack. After getting the projection, each channel was processed individually. The blue nuclei and red dots were segmented by filtering with a median filter, subtracting the background, enhancing the contrast with the Contrast Limited Adaptive Histogram Equalization plug-in, and finally applying a threshold to obtain the binary image and the regions of interest.

##### Bioluminescence resonance energy transfer (BRET)

HEK-293T cells growing in 6-well plates were transiently cotransfected with a constant amount of cDNA encoding the receptor fused to Rluc protein and with increasingly amounts of GFP-BiP-IR. To quantify protein-GFP expression, cells (20 μg total protein) were distributed in 96-well microplates (black plates with a transparent bottom) and the fluorescence was read in a Fluostar Optima fluorimeter (BMG Labtech) equipped with a high-energy xenon flash lamp using a 10 nm bandwidth excitation filter at 410 nm for protein-GFP reading. Protein-fluorescence expression was determined as fluorescence of the sample minus the fluorescence of cells expressing only the BRET donor. For BRET measurements, cells (20 μg of protein) were distributed in 96-well microplates (Corning 3600, White plates; Sigma) and BRET signal was collected 1 min after addition of 5 μm DeepBlueC (Invitrogen) using a Mithras LB 940 reader (Berthold Technologies) that allows the integration of the signals detected in the short-wavelength filter at 400 nm and the long-wavelength filter at 510 nm. To quantify receptor-Rluc expression, luminescence readings were also performed after 10 min of adding 5 μm DeepBlueC (Invitrogen). The net BRET is defined as [(long-wavelength emission)/(short-wavelength emission)] – Cf where Cf corresponds to [(long-wavelength emission)/(short-wavelength emission)] for the Rluc construct expressed alone in the same experiment. BRET is expressed as milli BRET units (mBU; net BRET × 1000). In BRET curves, BRET was expressed as a function of the ratio between fluorescence and luminescence (GFP/Rluc). To calculate maximal BRET from saturation curves, data were fitted using a nonlinear regression equation and assuming a single phase with GraphPad Prism software version 8.0.1. Each representative curve shown is the mean of three internal replicates.

##### Western blot and coimmunoprecipitation

Samples for Western blotting were prepared on ice-cold lysis buffer (50 mm Tris-HCl, 1 mm EDTA, 1 mm EGTA, 0.1% Triton X-100, 50 mm NaF, 10 mm Na-glycerophosphate, 5 mm Na-pyrophosphate, 1 mm Na-orthovanadate, pH 7.5). Cell lysates were clarified by centrifugation at 12,000 × *g* for 15 min (4°C), and total protein was quantified using the Bradford assay. Then, 5-20 µg aliquots of total protein, boiled for 5 min at 95°C and prepared in 5× Laemmli sample buffer, were resolved by using SDS-PAGE and transferred to PVDF membranes. Membranes were blocked with 5% defatted milk (w/v) or 5% BSA (w/v) in TBS-Tween-20 (0.1%) for 1 h and incubated overnight with the following antibodies and dilutions: anti-phospho-ERK1/2 (1:1000, CST, #9101), anti-ERK1/2 (1:1000, CST, #4696), anti-phospho-p70S6K (1:1000, CST, #9206), anti-phospho-CREB (1:1000, CST, #9198), anti-BiP (1:1000, Sigma-Aldrich, #G8918), anti-GFP (1:1000, Thermo Fisher Scientific, #MA5-15 256), anti-α-tubulin (1:10,000, Sigma-Aldrich, #T9026), anti-β-actin (1:10,000, Sigma-Aldrich, #A5441), anti-FLAG M2 (1:1000, Sigma-Aldrich, #F3165), anti-HA (1:1000, CST, #3724S), and anti-calnexin (1:1000, Santa Cruz Biotechnology, #SC-6465). All antibodies were prepared in TBS-Tween-20 (0.1%) with 5% BSA (w/v). Membranes were then washed 3 times with TBS-Tween-20 (0.1%), and HRP-labeled secondary antibodies, selected according to the species of origin of the primary antibodies (Sigma-Aldrich, #NA-931-1 and #NA-934V), were added for 1 h at a 1:5000 dilution in TBS-Tween-20 (0.1%) at room temperature. Finally, protein bands were detected by incubation with an enhanced chemiluminescence reagent (Bio-Rad), and densitometric analysis of the relative expression of the protein of interest versus the corresponding loading control was performed with ImageJ software. Western blot images were cropped for clarity. Electrophoretic migration of molecular weight markers is depicted on the left-hand side of each blot.

For coimmunoprecipitation experiments, 48 h after transfection, cells were lysed on ice-cold GST buffer (50 mm Tris-HCl, 10% glycerol v/v, 100 mm NaCl, 2 mm MgCl_2_, 1% v/v NP-40, pH 7.4), supplemented with protease inhibitors. Cell lysates were clarified by centrifugation at 12,000 × *g* for 15 min (4°C), and total protein was quantified with Bradford assay; 20 µg aliquots were collected to check for transfection levels (whole-cell lysates), and 1 mg of total protein was incubated with 20 µl of HA-agarose beads (Thermo Fisher Scientific, #26181) or FLAG M2 agarose beads (Sigma-Aldrich, #A2220) for 2-4 h at 4°C with a final protein concentration of 1 mg/ml. Beads were subsequently washed 3 times with lysis buffer and eluted with 30 µl of 2× Laemmli Sample Buffer without β-mercaptoethanol and 5 min of sample boiling; 10 µl of the elution was further analyzed by Western blotting as previously described. GFP immunoprecipitation was performed analogously, with a preclarification step on 30 µl of Protein A/G (GE Healthcare, *#*17061801), followed by overnight incubation of the remaining supernatant with 1 µg of anti-GFP antibody (produced in-home), and 2-4 h of incubation with 30 µl of Protein A/G mixture. The rest of the steps were identical to those mentioned above.

##### Dynamic mass redistribution (DMR)

The cell-signaling signature was determined using an EnSpire Multimode Plate Reader (PerkinElmer) by a label-free technology. Cellular mass movements induced on receptor activation were detected by illuminating the underside of the biosensor with polychromatic light and measured as changes in wavelength of the reflected monochromatic light that is a sensitive function of the index of refraction. The magnitude of this wavelength shift (herein measured in picometers) is directly proportional to the amount of DMR. Briefly, 24 h before the assay, cells were seeded at a density of 10,000 cells per well in 384-well sensor microplates with 30 μl growth medium and cultured for 24 h (37°C, 5% CO_2_) to obtain 70%-80% confluent monolayers. Previous to the assay, cells were washed twice with assay buffer (HBSS with 20 mm HEPES, pH 7.15) and incubated for 2 h in 30 μl per well of assay buffer with 0.1% DMSO in the reader at 24°C. Hereafter, the sensor plate was scanned, and a baseline optical signature was recorded before adding 10 μl of the test compound dissolved in assay buffer containing 0.1% DMSO. Then, DMR responses were monitored along time, and kinetic data were analyzed using EnSpire Workstation Software version 4.10. Each representative curve shown is the mean of three internal replicates.

##### Phosphoprotein array

Cells transfected with CB1R-GFP and BiP-IR (or control) plasmids were treated with WIN-55212-2 (100 nm final concentration) or vehicle (DMSO, 0.1% v/v final concentration) as described above for 5 and 15 min. Samples from two independent experiments were processed separately by using 350 µg of total protein per experimental condition, following the instructions of the Proteome Profiler Human Phospho-Kinase Array Kit (R&D Systems, Bio-techne, #ARY003C). Densitometric analysis of the relative phosphorylation levels versus the corresponding housekeeping controls and between WIN-55212-2/vehicle treatments was performed with ImageJ software and the Protein Array Analyzer toolset.

##### Cellular and subcellular fraction preparations

Membrane preparations for G-protein-coupling assays were obtained from HEK-293T-cell pellets or adult mouse-hippocampus tissue specimens. Frozen samples were thawed at 4°C and homogenized with a glass/Teflon grinder (IKA labortechnik), 10 strokes at maximum speed, in 30 volumes of homogenization buffer (250 mm sucrose, 50 mm Tris-HCl, 1 mm EGTA, 3 mm MgCl_2_, 1 mm DTT, pH 7.4). The homogenates were centrifuged at 1100 × *g* for 10 min at 4°C. The pellets were discarded, and the supernatants were recentrifuged at 40,000 × *g* for 10 min at 4°C. The resultant pellets were resuspended in 20 volumes of ice-cold centrifugation buffer (50 mm Tris-HCl, 1 mm EGTA, 3 mm MgCl_2_, 1 mm DTT, pH 7.4) with a glass stick and recentrifuged at 40,000 × *g* for 10 min at 4°C. The pellets obtained were then resuspended in 5 volumes of centrifugation buffer. Protein content was determined by the Bradford method. Finally, aliquots of 0.5, 1.0, and 2.0 mg protein were centrifuged at 21,000 × *g* for 15 min at 4°C. The supernatant layer was carefully discarded, and the pellets were stored at −80°C until assayed.

Total, cytosolic, and ER fractions from hippocampus, cortex, and striatum of the adult mouse brain were obtained by lysing the corresponding regions through sonication in 2 ml of ice-cold MTE buffer (270 mm D-mannitol, 10 mm Tris-HCl, 0.1 mm EDTA, pH 7.4). Tissue extracts were centrifuged (1400 × *g*, 10 min, 4°C), and the supernatant (total cell lysate) was recentrifuged (15,000 × *g*, 10 min, 4°C) to separate the pelleted mitochondrial crude fraction. Isolation of ER from cytosol was achieved by loading the sample in a sucrose gradient (2 m - 1.5 m - 1.3 m) and conducting an ultracentrifugation step (152,000 × *g*, 70 min, 4°C). The ER fraction appears as a band at the 1.5 m/1.3 m sucrose interphase, while the cytosolic fraction remains at the top of the tube. Both fractions were collected, in the case of the ER with the aid of a syringe with a 20G needle, and the ER fraction was further purified by an additional ultracentrifugation step (126,000 × *g*, 45 min, 4°C). The ER-containing pellet was resuspended in 100 µl of PBS and immediately frozen. Likewise, aliquots of total cell lysate and cytosolic fractions were collected throughout the process and immediately frozen. Samples were kept at −80°C for Western blot analysis.

Striatal, hippocampal, and cortical synaptosomes were isolated from adult CB_1_R-KO mice and CB1R-WT control littermates, plated on poly-L-lysine-covered coverslips, fixed in 4% PFA, and characterized as described previously (Martín et al., 2010). PLA assays were conducted as described above.

##### Antibody-capture [35S]GTPγS scintillation proximity assay

Specific activation of different subtypes of Gα protein subunits (Gα_i1_, Gα_i2_, Gα_i3_, Gα_o_, Gα_q/11_, Gα_s_, Gα_z_, and Gα_12/13_) was determined by using a homogeneous protocol of [^35^S]GTPγS scintillation proximity assay coupled to the use of the following antibodies: mouse monoclonal anti-Gα_i1_ (1:20, Santa Cruz Biotechnology, #sc-56536), rabbit polyclonal anti-Gα_i2_ (1:20; Santa Cruz Biotechnology, #sc-7276), rabbit polyclonal anti-Gαi3 (1:30, Antibodies on-line, #ABIN6258933), mouse monoclonal anti-Gα_o_ (1:40, Santa Cruz Biotechnology, #sc-393874), mouse monoclonal anti-Gα_q/11_ (1:20, Santa Cruz Biotechnology, #sc-515689), rabbit polyclonal anti-Gα_s_ (1:20, Santa Cruz Biotechnology, #sc-383), rabbit polyclonal anti-Gα_z_ (1:20, Santa Cruz Biotechnology, #sc-388), and rabbit polyclonal anti-Gα_12/13_ (1:20 Santa Cruz Biotechnology, sc-28 588). [^35^S]GTPγS binding was measured in 96-well isoplates (PerkinElmer Life Sciences) and a final volume of 200 μl containing 1 mm EGTA, 3 mm MgCl_2_, 100 mm NaCl, 0.2 mm DTT, 50 mm Tris-HCl, pH 7.4, 0.4 nm [^35^S]GTPγS, 10 µg of protein per well, and different concentrations of GDP (between 50 and 100 μm) depending on the Gα subunit subtype tested. At the end of the 2 h incubation period (at 30°C), 20 µl of 1% Igepal plus 0.1% SDS was added to each well, and plates were incubated at 22°C for 30 min with gentle agitation. The specific antibody for the Gα subunit of interest was then added to each well before an additional 90 min incubation period at room temperature. Polyvinyltoluene SPA beads coated with protein A (PerkinElmer) were then added (0.75 mg of beads per well), and plates were incubated for 3 h at room temperature with gentle agitation. Finally, plates were centrifuged (5 min at 1000 × *g*), and the bound radioactivity was detected on a MicroBeta TriLux scintillation counter (PerkinElmer). To determine their effect on [^35^S]GTPγS binding to the different Gα subunit subtypes in the different experimental conditions, a single submaximal concentration (10 μm) of WIN-55212-2 was used, either alone or in the presence of the CB_1_R antagonist O-2050 (10 μm) as control. Nonspecific binding was defined as the remaining [^35^S]GTPγS binding in the presence of 10 μm unlabeled GTPγS. For each Gα protein, specific [^35^S]GTPγS binding values were transformed to percentages of basal [^35^S]GTPγS binding values (those obtained in the presence of vehicle).

##### Determination of cAMP concentration

Homogeneous time-resolved fluorescence energy transfer assays were performed using the Lance Ultra cAMP kit (PerkinElmer). HEK-293T cells (1000 per well), growing in medium containing 50 μm zardeverine, were incubated in triplicate for 15 min in white ProxiPlate 384-well microplates (PerkinElmer) at 25°C with vehicle or WIN-55212-2 (100 nm final concentration) before adding vehicle or forskolin (0.5 μm final concentration) and incubating for 15 additional minutes. Fluorescence at 665 nm was analyzed on a PHERAstar Flagship microplate reader equipped with a homogeneous time-resolved fluorescence optical module (BMG Lab Technologies).

##### Animals

All the experimental procedures used were performed in accordance with the guidelines and approval of the Animal Welfare Committees of Universidad Complutense de Madrid and Comunidad de Madrid, as well as of Universitat de Barcelona and Generalitat de Catalunya, and in accordance with the directives of the Spanish Government and the European Commission. BiP^+/−^ (herein referred to as BiP-HET) mice were purchased from The Jackson Laboratory (#019549). We also used CB_1_R*^floxed/floxed^* (herein referred to as CB_1_R-floxed) mice, CB_1_R*^floxed/floxed;CMV-Cre^* (herein referred to as CB_1_R-KO) mice, conditional CB_1_R*^floxed/floxed;Nex1-Cre^* (herein referred to as Glu-CB_1_R-KO) mice, and conditional CB_1_R*^floxed/floxed;Dlx5/6-Cre^* (herein referred to as GABA-CB_1_R-KO) mice (Monory et al., 2006); as well as Stop-CB_1_R, Stop-CB_1_R*^EIIa-Cre^* (herein referred to as CB_1_R-RS) mice, conditional Stop-CB_1_R*^Nex1-Cre^* (herein referred to as Glu- CB_1_R-RS) mice, and conditional Stop-CB_1_R*^Dlx5/6-Cre^* (herein referred to as GABA-CB_1_R-RS) mice, to allow CB_1_R gene-expression rescue from a CB_1_R-null background (Ruehle et al., 2013; De Salas-Quiroga et al., 2015). Animal housing, handling, and assignment to the different experimental groups were conducted as described previously (Ruiz-Calvo et al., 2018). Adequate measures were taken to minimize pain and discomfort of the animals.

##### ISH histochemistry

For ISH histochemistry, 14-μm-thick coronal whole-brain tissue sections were obtained from adult C57BL/6 mice (Janvier Laboratories), cut on a microtome-cryostat (Microm HM500 OM), thaw-mounted on 3-aminopropyltriethoxysilane-coated slides (Sigma-Aldrich), and kept at −20°C until further processing. The oligonucleotides complementary to the mRNAs encoding BiP, CB_1_R, and GABAergic or glutamatergic markers are listed in Table 1. Oligonucleotides for each mRNA were labeled at their 3′-end by using [α-^33^P]dATP (3000 Ci/mmol, Hartmann Analytic). Labeled probes were purified on ProbeQuant G-50 Micro Columns (GE Healthcare). ISH histochemistry procedures were performed as described previously (Sanabra and Mengod, 2011). For autoradiography, hybridized sections were exposed to Biomax-MR (Kodak) films for 1-10 d at −70°C with intensifying screens. Double *in situ*-hybridized sections were processed as described previously (Reyes-Irisarri et al., 2007). They were exposed in the dark for 4-6 weeks at 4°C. Images from autoradiograms were obtained by using a Wild 420 macroscope (Leica Microsystems) equipped with a digital camera (DXM1200 F, Nikon) and ACT-1 Nikon software. Microphotography was performed with an Olympus BX51 Stereologic Microscope (Olympus) equipped with a digital camera (DP71, Olympus) or with a Carl Zeiss Axioplan microscope equipped with an Olympus XC50 digital camera. Figures were assembled using Adobe Photoshop (Adobe Systems). Only contrast and brightness were uniformly adjusted to optimize images.

**Table 1.** Oligonucleotides used for ISH histochemistry

Oligonucleotide designation	mRNA	Accession number	bp limits	Oligonucleotide sequence
mCB1/1	Cannabinoid receptor 1	U22948.1	186-230	GATGGTACGGAAGGTGGTATCTGCAAGGCCGTCTAAGATCGACTT
mCB1/2	Cannabinoid receptor 1	U22948.1	556-600	ATAGCACCAGCAGGTTCTCCAGAACCGTGAAGGTGCCCAGGGTGA
mCB1/3	Cannabinoid receptor 1	U22948.1	1556-1601	CAGAGCCTCGGCAGACGTGTCTGTGGACACAGACATGGTCACCTT
mGRP78/1	78 kDa glucose-regulated protein (BiP)	D78645.1	121-165	TCTTGTCCTCCTCCTCGGCCCGCACCGCGCCCAGCAGCAGCAACG
mGRP78/2	78 kDa glucose-regulated protein (BiP)	D78645.1	1262-1306	ACACCAGCCTGGACAGCGGCACCATAGGCTACAGCCTCATCGGGG
mGRP78/3	78 kDa glucose-regulated protein (BiP)	D78645.1	1996-2040	ATGTATCCTCTTCACCAGTTGGGGGAGGGCCTCCACTTCCATAGA
rmGAD65/1	Glutamic acid decarboxylase 65	NM_008078.2	421-465	CTTGTTTCCGATGCCGCCCGTGAACTTTTGGGCCACCTGGCACCA
rmGAD65/2	Glutamic acid decarboxylase 65	NM_008078.2	776-820	GCGTCAAAATTTCCTCCAGATTTTGCGGTTGGTCTGCCAATTCCC
rGAD/5	Glutamic acid decarboxylase 67	M76177.1	1601-1654	ATAGAGGTATTCAGCCAGCTCCAGGCATTTGTTGATCTGATTTTCAAATCCCAC
rVGluT1/1	Vesicular GluT1 transporter	NM_053859.1	127-171	CAGGGCGCGCCCCGCCAGCTTCCGAAACTCCTCCTGCCGGAACTC
rVGluT1/2	Vesicular GluT1 transporter	NM_053859.1	1756-1800	GTCCCGGACAGGGGGTGGGGGCCTTGGAGGCTGAACTGTGCTGTG

##### Behavioral tests

Adult male mice (3- to 4-month-old) were injected intraperitoneally with vehicle (2% v/v DMSO in 1:18 v/v Tween-80/saline solution) or 10 mg/kg THC (THC Pharm). The “cannabinoid tetrad” was assessed, starting 30 min after injection, following standard guidelines (Metna-Laurent et al., 2017). First, the open-field test was conducted for 10 min in an arena of 70 × 70 cm. To evaluate anxiety-like behaviors, the number of entries of the animal into the central part of the arena (25 × 25 cm) relative to total ambulation was assessed, one entry being counted when the animal had placed at least both forelimbs in the square. Next, analgesia was assessed as the latency to paw licking in the hotplate paradigm at a constant temperature of 52°C. Then, for the catalepsy test, the animal was placed with both forelimbs leaning on a bar situated at a height of 3.5 cm. Immobility was considered maximal when the animal exceeded 60 s of immobility, and null when the immobility time was lower than 5 s. In all cases, three attempts were performed, and the maximal immobility time was selected as the representative value. Finally, body temperature was measured with a rectal thermometer and compared with the basal, pre-injection value.

The elevated plus maze test was evaluated 4 h after acute intraperitoneal injection of vehicle or THC (10 mg/kg). The maze consisted of a cross-shaped plastic device with two opposite open arms (30-cm-long, 5-cm-wide) and two opposite closed arms (30-cm-long, 5-cm-wide, 16-cm-tall walls), connected by a central structure (5 × 5 cm), and elevated 50 cm from the floor. Each mouse was placed in the center of the maze, facing one of the open arms, and the exploratory behavior of the animal was video-recorded for 5 min. The number and duration of entries were measured separately for the open arms and the closed arms. One arm entry was registered when the animal had placed both forepaws in the arm.

In all cases, animals were assigned randomly to the different treatment groups, and all experiments were performed in a blinded manner for genotype and pharmacological treatment. All tests were video-recorded for subsequent blinded analysis using Smart3.0 version 3.00.6 Software (Panlab).

##### Experimental design and statistical analyses

Unless otherwise specified, data are presented as mean ± SEM. Statistical comparisons were conducted by one-way or two-way ANOVA with Tukey's *post hoc* test, or by Student's *t* test, as indicated in each case. All datasets were tested for normality (Kolmogorov–Smirnov's test) and homoscedasticity (Levene's test) before analysis. For clarity, only *p* values < 0.05 were considered statistically significant. The sample size for each experiment was estimated on the basis of previous studies conducted by our laboratories using similar protein-interaction, cell-culture, brain-sample, and motor-behavior approaches. Subsequent power analysis was conducted for each parameter by using IBM SPSS software. The number of biological replicates (e.g., number of mice, number of cell cultures) is provided in the corresponding figure legends. The number of technical replicates (e.g., number of Y2H assays, number of incubations within each cell culture, number of sections microscopically analyzed per mouse brain, number of behavioral trials per mouse) is provided in the corresponding figure legends or in the corresponding Materials and Methods subsections. All the experiments conducted with animals are presented as dot plots. Graphs and statistics were generated by GraphPad Prism version 8.0.1.

## Results

### BiP interacts with CB_1_R *in vitro*

**Figure 1.**
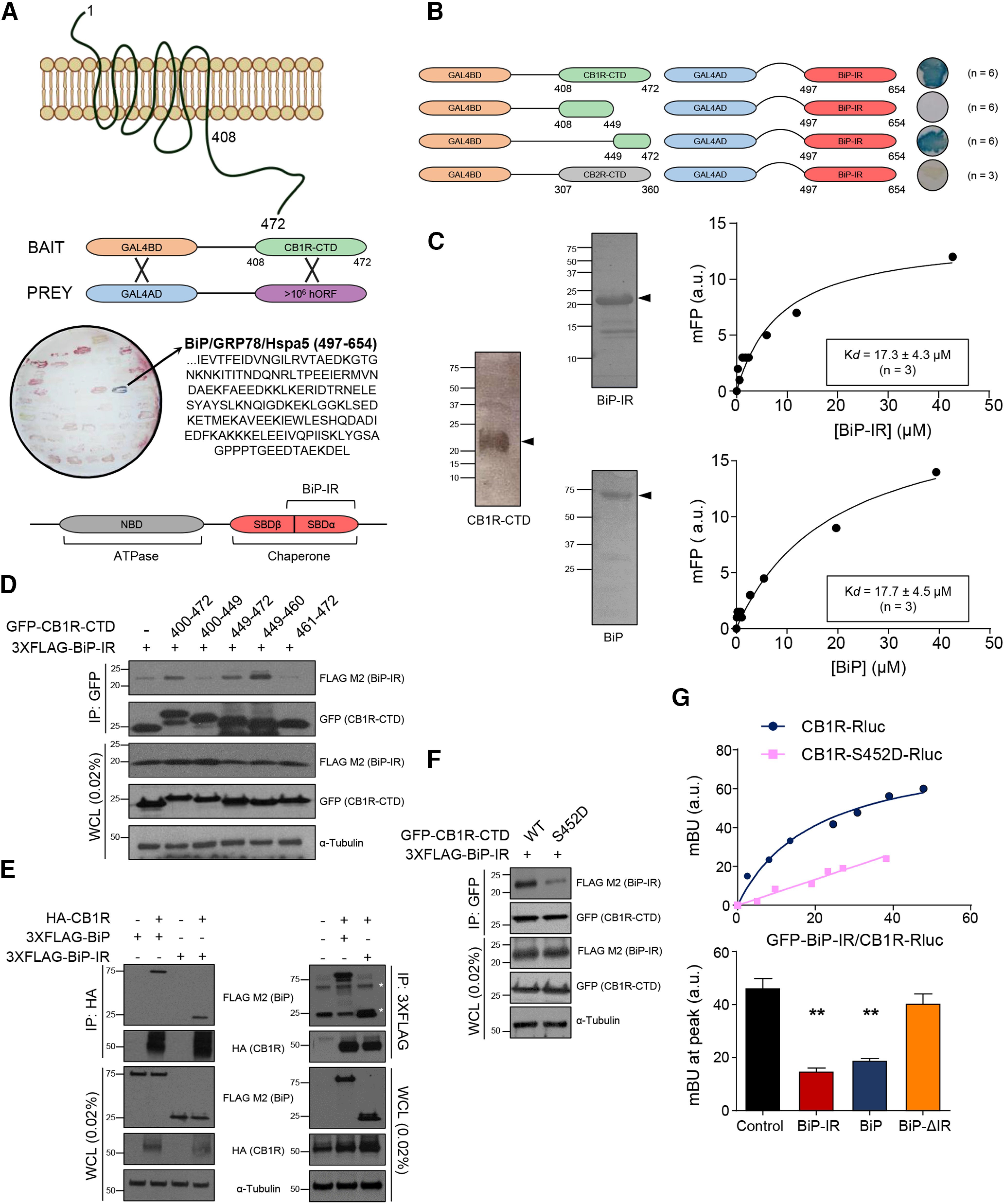
BiP interacts with CB_1_R *in vitro*. ***A***, Scheme of the Y2H experiment using CB_1_R-CTD (amino acids 408-472) as bait and a human cDNA library (>10^6^ clones) as prey. One cDNA clone (stained in blue) contained BiP/GRP78/Hspa5 amino acids 497-654 (BiP-IR). Diagram represents the main structural domains of BiP/GRP78/Hspa5 is shown. ***B***, Scheme of the Y2H experiment using fragments of CB_1_R-CTD or CB_2_R-CTD as bait and BiP-IR as prey. ***C***, Fluorescence polarization-based protein–protein binding experiments using 5-IAF-labeled CB_1_R-CTD and increasing amounts of unlabeled BiP-IR (top) or BiP (bottom). A representative experiment, including the gels of the purified proteins, is shown (*n* = 3). ***D***, Coimmunoprecipitation experiments in HEK-293T cells expressing fragments of GFP-CB_1_R-CTD and 3xFLAG-BiP-IR. Immunoprecipitation (IP) was conducted with anti-GFP antibody. WCL, Whole-cell lysate. A representative experiment is shown (*n* = 3). ***E***, Coimmunoprecipitation experiments in HEK-293T cells expressing HA-CB_1_R and 3xFLAG-BiP or 3xFLAG-BiP-IR. IP was conducted with anti-HA antibody (left) or anti-FLAG antibody (right). Asterisk indicates immunoglobulin heavy and light chains. A representative experiment is shown (*n* = 3). ***F***, Coimmunoprecipitation experiments in HEK-293T cells expressing GFP-CB_1_R-CTD WT or an S452D point mutant form, along with 3xFLAG-BiP-IR. IP was conducted with anti-GFP antibody. A representative experiment is shown (*n* = 3). ***G***, BRET experiments in HEK-293T cells expressing CB1R-Rluc or CB1R-S452D-Rluc and increasing amounts of GFP-BiP-IR (top; a representative experiment is shown; *n* = 3), together or not with nontagged versions of BiP, BiP-IR, or BiP-ΔIR as competitors (bottom). **p < 0.01 from control vector by one-way ANOVA with Tukey's multiple comparisons test (*n* = 3).

To identify new CB_1_R-interacting intracellular proteins, we challenged the receptor's CTD (amino acids 408-472) to a cDNA library containing >10^6^ different clones by means of a Y2H system. One particular cDNA clone, comprising amino acids 497-654 of the protein BiP (hereafter “BiP-interacting region” [BiP-IR]), provided an unequivocally positive outcome (Fig. 1*A*). BiP, also known as GRP78 or Hspa5, belongs to the highly conserved Hsp70 family of molecular chaperones. These proteins consist of two different domains: an *N*-terminal nucleotide-binding domain with ATPase activity, and a *C*-terminal substrate-binding domain (SBD). The SBD, in turn, is composed of a β-sandwich domain (SBDβ) and an α-helical lid (SBDα), which are interlinked by a hydrophobic stretch (Wieteska et al., 2017). It is generally believed that ATP-assisted, BiP-mediated protein refolding proceeds when hydrophobic peptides bind to a conserved groove in the SBDβ domain of BiP. Conversely, here, we found that CB_1_R-CTD interacts essentially with the lid domain in the absence of the groove. Specifically, according to the reported structures (Yang et al., 2015, 2017), BiP-IR would span the entire SBDα and two strands of the SBDβ (Fig. 1*A*, bottom diagram).

We next aimed to validate the molecular specificity of the interaction between CB_1_R-CTD and BiP-IR. First, by using directed Y2H assays, we delimitated the BiP-IR-binding site to a restricted 23 amino-acid stretch (residues 449-472) at the edge of CB_1_R-CTD (Fig. 1*B*). Second, we found that the CTD of CB_2_R, the GPCR with the highest sequence homology to CB_1_R, did not bind BiP-IR (Fig. 1*B*). Third, as the phosphorylation state of specific S and T residues in the CTD of a GPCR can determine its interaction with intracellular proteins, we challenged BiP-IR to every possible single phosphomimetic mutant (S/T → D) within CB_1_R-CTD, and found that only the S452D point mutation, which is precisely located in the last 23 amino-acid portion of CB_1_R, impaired the association (Table 2). Fourth, we expressed recombinant CB_1_R-CTD, BiP-IR, and BiP, and found that BiP and BiP-IR bind CB_1_R-CTD with a similar high affinity, as measured by fluorescence polarization-based protein–protein binding assays (Fig. 1*C*).

**Table 2.** Effect of CB_1_R-CTD phosphomimetic mutants on CB_1_R-BiP interaction*^a^*

Bait plasmid	Prey plasmid	Interaction
pGBT9 CB_1_R-CTD-S410D	pACT2 BiP-IR	+ (*n* = 3)
pGBT9 CB_1_R-CTD-S414D	pACT2 BiP-IR	+ (*n* = 3)
pGBT9 CB_1_R-CTD-T418D	pACT2 BiP-IR	+ (*n* = 3)
pGBT9 CB_1_R-CTD-S425D	pACT2 BiP-IR	+ (*n* = 3)
pGBT9 CB_1_R-CTD-S429D	pACT2 BiP-IR	+ (*n* = 3)
pGBT9 CB_1_R-CTD-S441D	pACT2 BiP-IR	+ (*n* = 3)
pGBT9 CB_1_R-CTD-S448D	pACT2 BiP-IR	+ (*n* = 3)
pGBT9 CB_1_R-CTD-S452D	pACT2 BiP-IR	− (*n* = 3)
pGBT9 CB_1_R-CTD-T453D	pACT2 BiP-IR	+ (*n* = 3)
pGBT9 CB_1_R-CTD-T460D	pACT2 BiP-IR	+ (*n* = 3)
pGBT9 CB_1_R-CTD-S462D	pACT2 BiP-IR	+ (*n* = 3)
pGBT9 CB_1_R-CTD-S464D	pACT2 BiP-IR	+ (*n* = 3)
pGBT9 CB_1_R-CTD-T465D	pACT2 BiP-IR	+ (*n* = 3)
pGBT9 CB_1_R-CTD-T467D	pACT2 BiP-IR	+ (*n* = 3)
pGBT9 CB_1_R-CTD-S468D	pACT2 BiP-IR	+ (*n* = 3)

*^a^*Scheme of the Y2H experiment using every possible single phosphomimetic mutant (S/T→D) within CB_1_R-CTD as bait, and BiP-IR as prey. Only one clone abrogated the interaction (CB_1_R-CTD-S452D).

**Figure 2.**
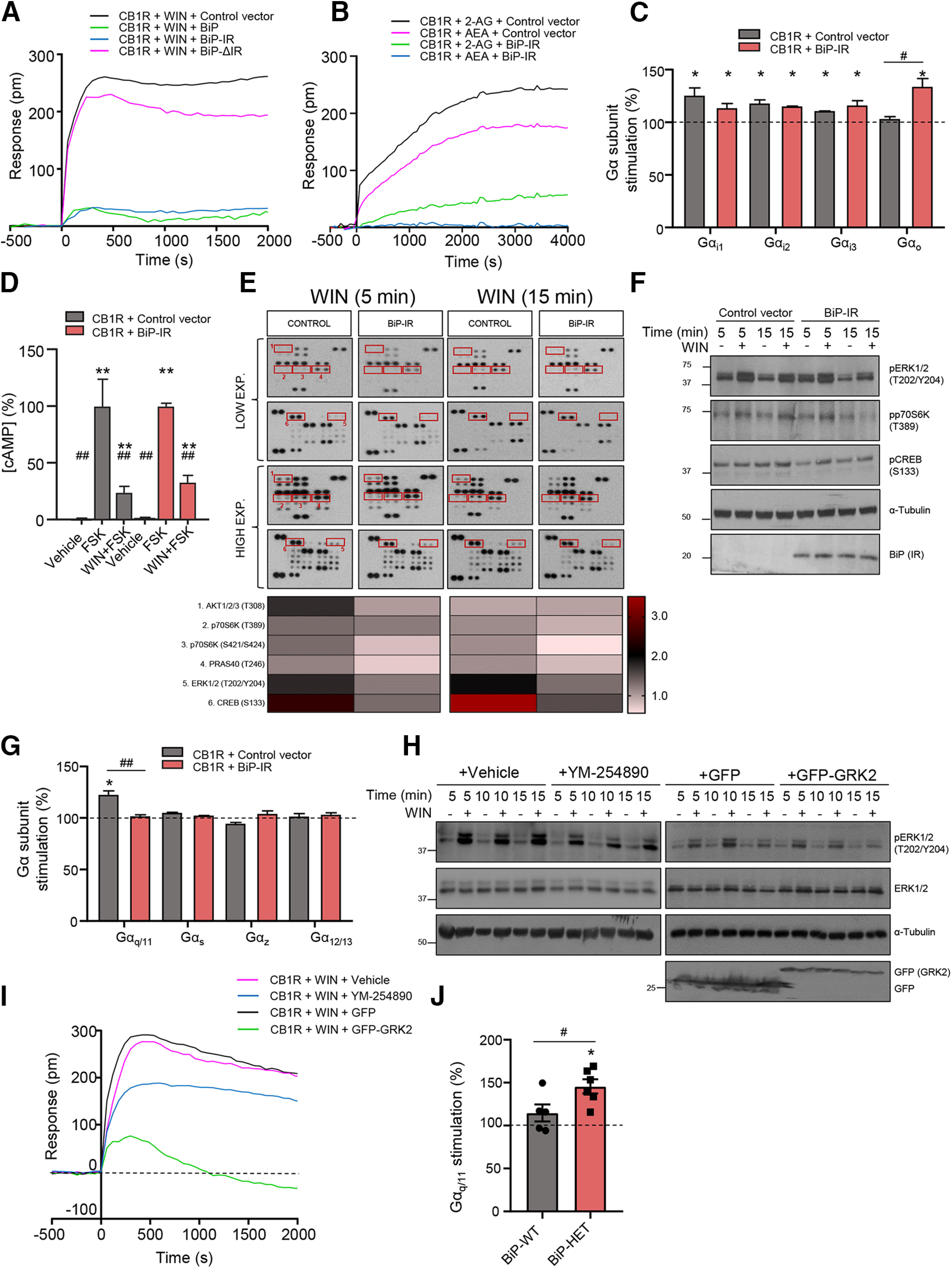
BiP modulates CB_1_R-evoked signaling. ***A***, DMR experiments in HEK-293T cells expressing CB_1_R, together or not with BiP, BiP-IR, or BiP-ΔIR, and incubated with WIN-55212-2 (100 nm). A representative experiment is shown (*n* = 3). ***B***, DMR experiments in HEK-293T cells expressing CB_1_R, together or not with BiP-IR, and incubated endocannabinoid (10 μm; 2-AG, 2-arachidonoylglycerol; AEA, anandamide). A representative experiment is shown (*n* = 3). ***C***, Coupling of CB_1_R to Gα_i/o_ proteins in membrane extracts from HEK-293T cells expressing CB_1_R, together or not with BiP-IR. *p < 0.05 from basal (dashed line), or ^#^*p* < 0.05 from control vector; one-sample Student's *t* test or unpaired Student's *t* test, respectively (*n* = 3). ***D***, cAMP concentration in HEK-293T cells expressing CB_1_R, together or not with BiP-IR. Cells were incubated first for 15 min with vehicle or WIN-55212-2 (100 nm), and then for 15 min with vehicle or forskolin (FSK; 500 nm). **p < 0.01 from vehicle, or ^##^*p* < 0.01 from FSK alone; two-way ANOVA with Tukey's multiple comparisons test (*n* = 3). ***E***, HEK-293T cells expressing CB_1_R, together or not with BiP-IR, were incubated for 5 or 15 min with vehicle or WIN-55212-2 (100 nm), and cell extracts were blotted on a phosphoprotein array. Two different times of membrane exposure are shown to allow an appropriate visualization of the main proteins affected (framed spots). A representative experiment is shown (*n* = 2; membranes from vehicle-treated cells are omitted for clarity). Heat map represents values of mean fold-activation by WIN-55212-2 over vehicle. ***F***, Validation of some of the phosphoarray hits by conventional Western blotting in the same cell extracts used in ***D***. A representative experiment is shown (*n* = 2). ***G***, Coupling of CB_1_R to non-Gα_i/o_ Gα proteins in membrane extracts from HEK-293T cells expressing CB_1_R, together or not with BiP-IR. *p < 0.05 from basal (dashed line), or ^##^*p* < 0.01 from control vector; one-sample Student's *t* test or unpaired Student's *t* test, respectively (*n* = 3). ***H***, Western blotting of phospho-ERK in HEK-293T cells expressing CB_1_R, and incubated for 5, 10, or 15 min with vehicle or WIN-55212-2 (100 nm). Top, Cells were preincubated for 30 min with vehicle or YM-254890 (1 μm). Bottom, Cells coexpressed control vector (GFP) or Gα_q/11_ dominant-negative vector (GFP-GRK2). A representative experiment is shown (*n* = 3). ***I***, DMR experiments in HEK-293T cells expressing CB_1_R under the same experimental conditions as in ***G***. A representative experiment is shown (*n* = 3). ***J***, Coupling of CB_1_R to Gα_q/11_ protein in hippocampal extracts from 3- to 4-month-old BiP^+/+^ (BiP-WT) and BiP^+/−^ (BiP-HET) mice. *p < 0.05 from basal (dashed line), or ^#^*p* < 0.05 from BiP-WT group; one-sample Student's *t* test or unpaired Student's *t* test, respectively (*n* = 5 or 6 mice per group).

We subsequently conducted experiments in HEK-293T cells. First, coimmunoprecipitation studies showed that (1) CB_1_R-CTD, and specifically its 449-460 amino-acid stretch, was sufficient to bind BiP-IR (Fig. 1*D*); (2) full-length CB_1_R also interacted with both BiP and BiP-IR (Fig. 1*E*); and (3) BiP-IR exhibited little association with the S452D point mutant of CB_1_R-CTD (Fig. 1*F*). Second, BRET experiments conducted with an Rluc-tagged version of CB_1_R also supported the protein–protein interaction (Fig. 1*G*, top), and adding non–GFP-tagged versions of BiP as competitors decreased the BRET peak only when the BiP-IR was present (Fig. 1*G*, bottom) (*n* = 3 experiments; BiP-IR vs control: *F*_(3,30)_ = 28.20, *p* < 0.0001, ANOVA; BiP vs control: *F*_(3,30)_ = 28.20, *p* < 0.0001, ANOVA; BiP-ΔIR vs control: *F*_(3,30)_ = 28.20, *p* = 0.3648, ANOVA). Moreover, there was no overt binding between GFP-BiP-IR and CB1R-Rluc when the S452D single mutation was introduced in the receptor (Fig. 1*G*, top).

Together, these data show that BiP interacts specifically with CB_1_R *in vitro*, both in purified-protein assays and in HEK-293T cells.

### BiP modulates CB_1_R-evoked signaling

**Figure 3.**
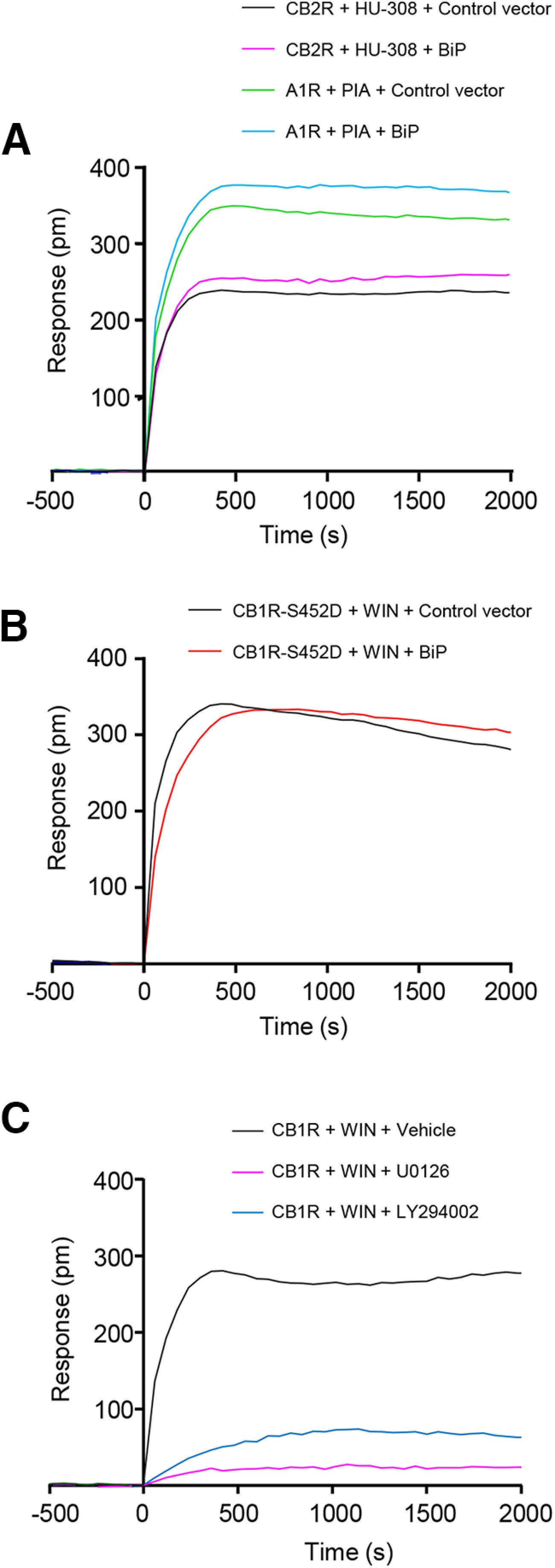
Controls of specificity of the CB_1_R-BiP DMR experiments. ***A***, DMR experiments in HEK-293T cells expressing CB_2_R, together or not with BiP, and incubated with the CB_2_R-selective agonist HU-308 (100 nm); or in HEK-293T cells expressing A_1_R, together or not with BiP, and incubated with the A_1_R-selective agonist PIA (50 nm). A representative experiment is shown (*n* = 3). ***B***, DMR experiments in HEK-293T cells expressing CB_1_R-S452D, together or not with BiP, and incubated with WIN-55212-2 (100 nm). A representative experiment is shown (*n* = 3). ***C***, DMR experiments in HEK-293T cells expressing CB_1_R and incubated with WIN-55212-2 (100 nm) plus vehicle or U0126 (5 μm) or LY294002 (5 μm). A representative experiment is shown (*n* = 3).

DMR is a powerful tool to assess the overall signal triggered by the agonist-evoked activation of a particular receptor in living cells (Fang et al., 2007). Indeed, we and others have previously used DMR to investigate CB_1_R-evoked signaling (Viñals et al., 2015; Moreno et al., 2018; Navarro et al., 2020). Here, by using HEK-293T cells expressing CB_1_R, we found a well-defined and saturating curve after adding the cannabinoid receptor-selective agonist WIN-55212-2 (Fig. 2*A*). Of note, coexpression of full-length BiP led to a strong inhibition of CB_1_R signaling (Fig. 2*A*) but did not alter the agonist-evoked response of two other G_i/o_-coupled receptors (CB_2_R and adenosine A_1_ receptor) that were used as controls (Fig. 3*A*). The effect of BiP on CB_1_R relied selectively on BiP-IR, as expressing this region rendered a comparable inhibition, and no change was found with BiP-ΔIR (Fig. 2*A*). This effect was again subverted when the S452D point mutation was inserted in CB_1_R (Fig. 3*B*), and was also evident, although with a slower kinetics, when the endocannabinoids anandamide and 2-arachidonoylglycerol were used as receptor agonists (Fig. 2*B*). Given the similar behavior of full-length BiP and BiP-IR, we used only BiP-IR for further signaling experiments.

CB_1_R activation modulates multiple signaling pathways, with cAMP/PKA, ERK, and PI3K/Akt/mTORC1 being the best characterized (Pertwee et al., 2010; Nogueras-Ortiz and Yudowski, 2016). We thus aimed to dissect in detail the inhibitory effect of BiP-IR on CB_1_R overall signaling observed in DMR assays. First, we found that BiP-IR did not alter markedly the archetypical Gα_i/o_-coupling profile of CB_1_R (Fig. 2*C*) (*n* = 3 experiments; Gα_i1_-control vs Gα_i1_-BiP-IR: *t*_(25)_ = 1.730, *p* = 0.0959, *t* test; Gα_i2_-control vs Gα_i2_-BiP-IR: *t*_(14)_ = 0.2886, *p* = 0.7771, *t* test; Gα_i3_-control vs Gα_i3_-BiP-IR: *t*_(18)_ = 0.5927, *p* = 0.5607, *t* test; Gα_o_-control vs Gα_o_-BiP-IR: *t*_(27)_ = 4.950, *p* < 0.0001, *t* test), nor affected the WIN-55212-2-evoked reduction of forskolin-augmented cAMP concentration (Fig. 2*D*) (*n* = 3 experiments; control vs BiP-IR: vehicle, *F*_(2,12)_ = 45.98, *p* > 0.9999, ANOVA; forskolin, *F*_(2,12)_ = 45.98, *p* > 0.9999, ANOVA; WIN-55212-2 + forskolin, *F*_(2,12)_ = 45.98, *p* = 0.9893, ANOVA). Next, we analyzed the phosphorylation (activation) state of major cellular protein kinases by using a phosphoprotein array. HEK-293T cells were transfected with the same constructs used in the aforementioned DMR assays, and subsequently treated with vehicle or WIN-55212-2. Among the different pathways activated by the cannabinoid, BiP-IR preferentially hampered the Akt/mTORC1 pathway (as inferred from Akt1/2/3-T308, PRAS40-T246, and p70S6K-T389 phosphorylation) and the ERK pathway (as inferred from ERK1/2-T202/Y204 phosphorylation) (Fig. 2*E*). The WIN-55212-2-mediated activation of CREB, an archetypical convergent substrate of the Akt/mTORC1 and ERK pathways, was also inhibited by BiP-IR (as inferred from CREB-S133 phosphorylation). We confirmed this BiP-mediated inhibition of CB_1_R-evoked signaling by analyzing pERK1/2-T202/Y204, pp70S6K-T389, and pCREB-S133 with conventional Western blotting (Fig. 2*F*). Accordingly, the PI3K inhibitor LY294002 and the MEK1 inhibitor U0126 blunted the WIN-55212-2-evoked DMR signal (Fig. 3*C*).

To study how BiP selectively alters CB_1_R-mediated signaling independently of Gα_i/o_ proteins, we evaluated the coupling of the receptor to non-Gα_i/o_ G-proteins. Of note, we found that CB_1_R also coupled to Gα_q/11_, and this association was impaired by BiP-IR (Fig. 2*G*) (*n* = 3 experiments; Gα_q/11_-control vs Gα_q/11_-BiP-IR: *t*_(26)_ = 3.238, *p* = 0.0033, *t* test; Gα_s_-control vs Gα_s_-BiP-IR: *t*_(8)_ = 0.2220, *p* = 0.8299, *t* test; Gα_z_-control vs Gα_z_-BiP-IR: *t*_(8)_ = 0.9241, *p* = 0.3825, *t* test; Gα_12/13_-control vs Gα_12/13_-BiP-IR: *t*_(18)_ = 0.3941, *p* = 0.6981, *t* test). Moreover, WIN-55212-2-mediated ERK activation was mitigated by either pharmacological blockade of Gα_q/11_ (with the drug YM-254890) or genetic interference of Gα_q/11_ signaling (with a dominant-negative GFP-GRK2 construct) (Andradas et al., 2016) (Fig. 2*H*). Likewise, YM-254890 and dominant-negative Gα_q/11_ reduced the WIN-55212-2-evoked DMR response (Fig. 2*I*). We next analyzed the coupling of CB_1_R to Gα_q/11_ in hippocampal extracts from adult BiP^+/−^ (hereafter BiP-HET) and BiP^+/+^ (hereafter BiP-WT) mice [very early embryonic lethality occurs in BiP^−/−^ mice (Luo et al., 2006).] In line with the aforementioned data from HEK-293T cells, CB_1_R showed a preference for Gα_q/11_ coupling in BiP-HET mice compared with their BiP-WT littermates (Fig. 2*J*) [BiP-WT (*n* = 5 mice) vs BiP-HET (*n* = 6 mice): *t*_(2)_ = 7.268, *p* = 0.0184, *t* test].

Together, these data show that BiP-IR affects CB_1_R-evoked signaling through the selective attenuation of an “alternative” Gα_q/11_ protein-driven module, while leaving the “classical” Gα_i/o_ protein-driven module essentially unaffected.

### CB_1_R-BiP complexes reside on GABAergic terminals of the mouse brain

**Figure 4.**
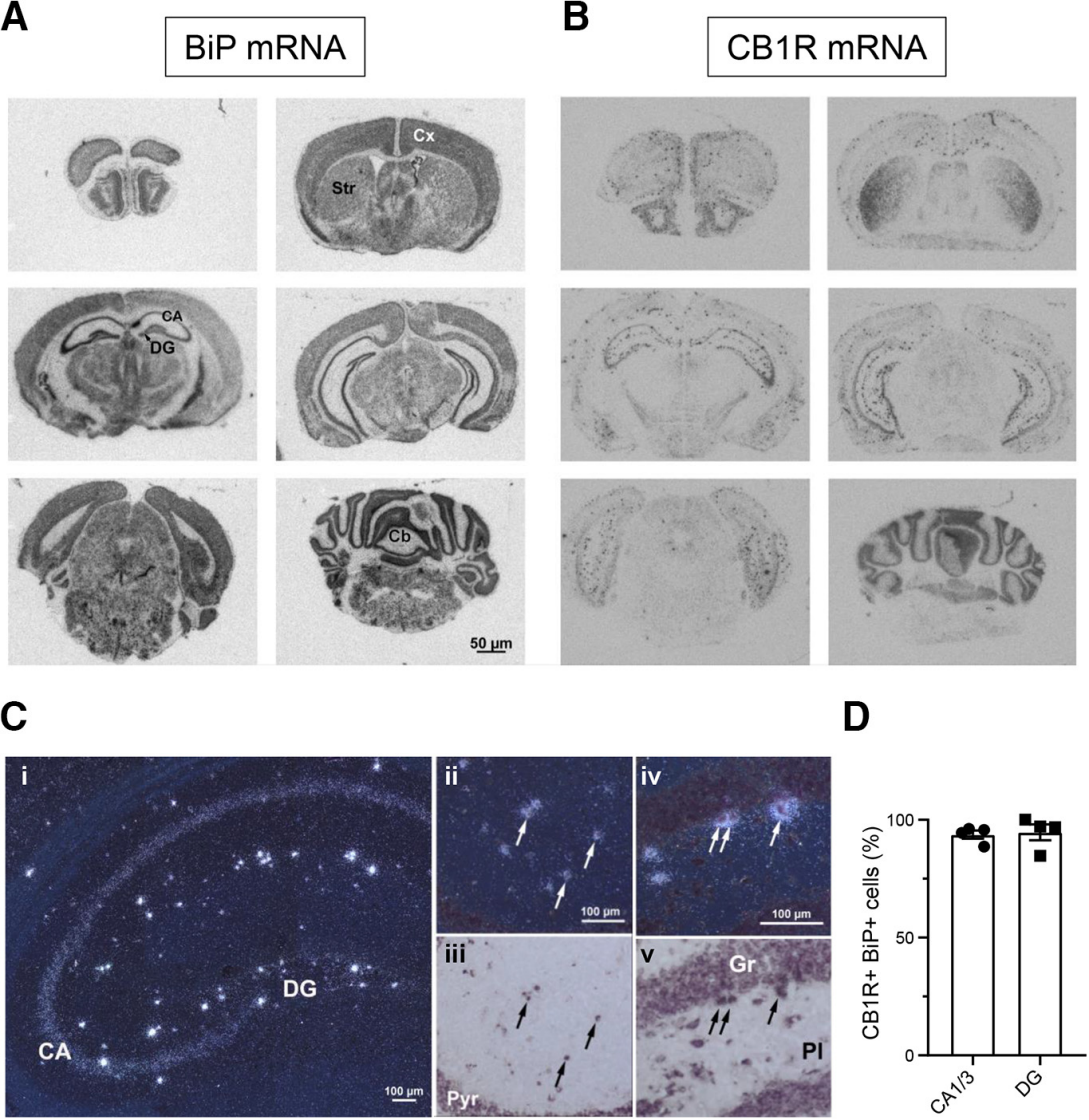
Expression of BiP and CB_1_R mRNA in the mouse brain. ***A***, ***B***, Representative autoradiographic images of coronal sections from adult mouse brain showing the mRNA hybridization pattern of BiP (***A***) and CB_1_R (***B***). CA, Cornu ammonis; DG, dentate gyrus; Str, striatum; Cx, cortex; Cb, cerebellum. ***C***, Distribution of CB_1_R mRNA in the mouse hippocampus. ***Ci***, Representative dark field image from a section hybridized with ^33^P-labeled oligonucleotide probes for CB_1_R mRNA. A positive signal is evident as clusters/accumulation of bright silver grains. Note the moderate signal on the pyramidal cell layer of CA and the very intense signal on scattered cells in the various hippocampal layers. ***Cii***, ***Ciii***, Colocalization of CB_1_R mRNA and BiP mRNA in cells of the stratum radiatum and stratum lacunosum moleculare of CA. Pyr, Pyramidal cell layer of CA. ***Civ***, ***Cv***, Colocalization of CB_1_R mRNA and BiP mRNA in cells of the polymorphic layer (Pl). Gr, Granular cell layer. Sections were hybridized with ^33^P-labeled probes for CB_1_R mRNA (signal visualized as clusters of bright silver grains in dark field images) and with digoxigenin-labeled probes for BiP mRNA (signal visualized as dark precipitate in bright field images). Arrows point to some double-labeled cells. ***D***, Quantification of CB_1_R mRNA-positive cells that coexpress BiP mRNA (*n* = 4 mice per group).

**Figure 5.**
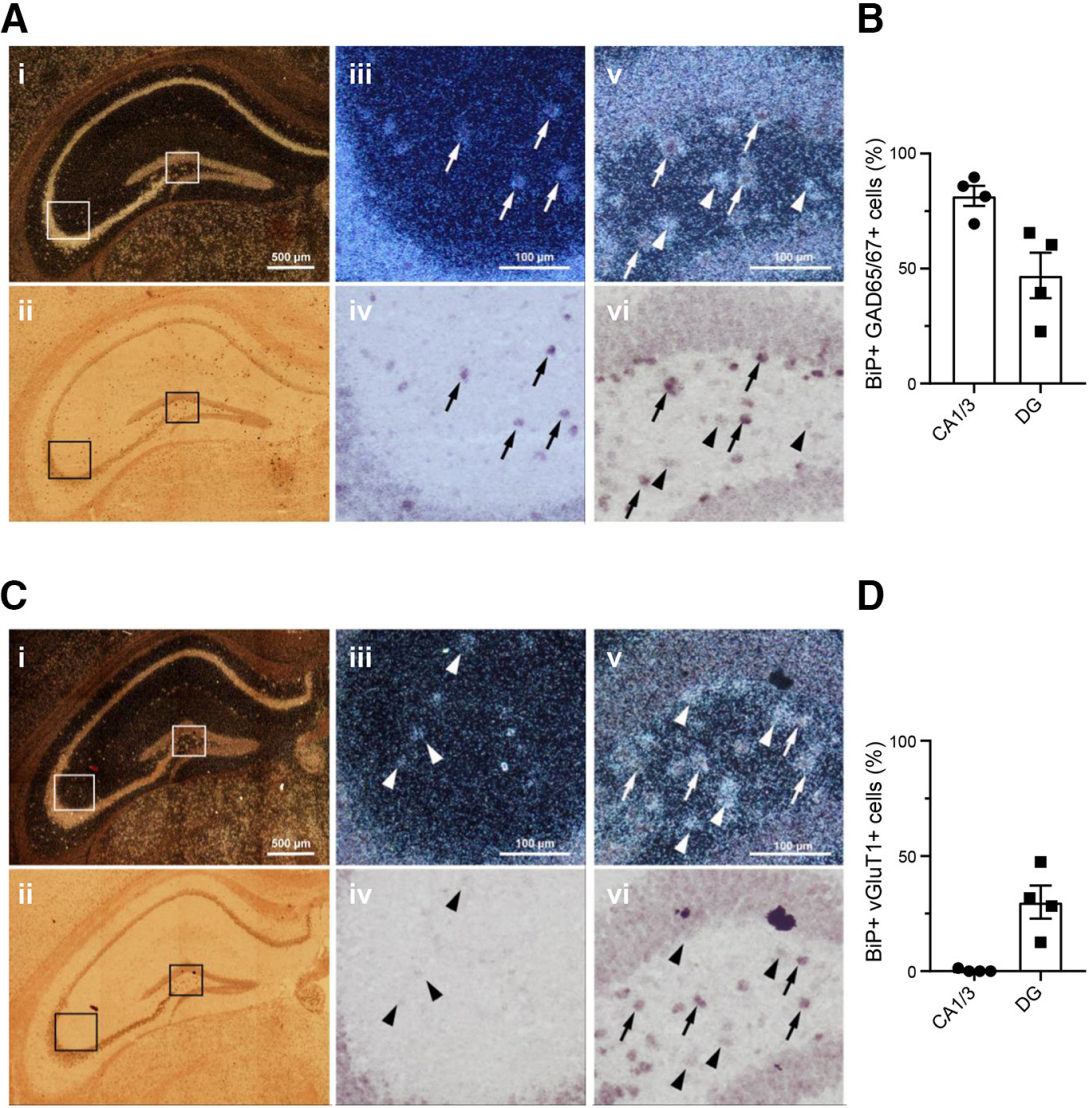
Colocalization of BiP mRNA with GAD65/67 or vGlut1 mRNA in the mouse hippocampus. ***A***, Representative mosaic superimages of sections from the adult mouse hippocampus that were hybridized with ^33^P-labeled probes for BiP mRNA (signal visualized as clusters/accumulation of bright silver grains in the dark field image ***Ai***) and with a mixture of digoxigenin-labeled probes for GAD65 and GAD67 mRNAs (labeled cells showing dark precipitate in the bright field image ***Aii***). Higher-magnification images of cornu ammonis (CA; ***Aiii***, ***Aiv***) and dentate gyrus (DG; ***Av***, ***Avi***) are shown. Arrows point to some double-labeled cells. Arrowheads point to some cells that express BiP mRNA but not GAD65/67 mRNA. ***B***, Quantification of BiP mRNA-positive cells that coexpress GAD65/67 mRNA (*n* = 4 mice per group). ***C***, Representative mosaic superimages of sections from the adult mouse hippocampus that were hybridized with ^33^P-labeled probes for BiP mRNA (signal visualized as clusters/accumulation of bright silver grains in the dark field image ***Ci***) and with digoxigenin-labeled probes for vGluT1 mRNA (labeled cells showing dark precipitate in the bright field image ***Cii***). Higher-magnification images of CA (***Ciii***, ***Civ***) and DG (***Cv***, ***Cvi***) are shown. Arrows point to some double-labeled cells. Arrowheads point to some cells that express BiP mRNA but not vGluT1 mRNA. ***D***, Quantification of BiP mRNA-positive cells that coexpress vGluT1 mRNA (*n* = 4 mice per group).

It is well established that CB_1_R resides largely on terminals of GABAergic neurons (Marsicano and Lutz, 1999; Katona and Freund, 2008). However, the precise neurochemical phenotype of BiP-expressing cells remains unclear (compare Jin et al., 2018). Hence, we analyzed the expression of BiP mRNA in GABAergic versus glutamatergic neurons by ISH histochemistry. BiP mRNA was localized throughout the mouse brain (Fig. 4*A*), showing a more ubiquitous expression pattern than CB_1_R mRNA (Fig. 4*B*). Of note, nearly all the hippocampal high CB_1_R mRNA-expressing cells were also positive for BiP mRNA [93.7 ± 1.7% in the CA1/3 areas and 94.6 ± 3.4% in the dentate gyrus (DG); *n* = 4 mice, *t*_(6)_ = 0.2487, *p* = 0.8119, *t* test] (Fig. 4*C*,*D*). In the CA1/3 hippocampal areas, as reported for CB_1_R mRNA (Marsicano and Lutz, 1999), BiP mRNA showed a high colocalization with GAD65/67 mRNA (81.6 ± 4.4% of the BiP-positive cells coexpressed GAD65/67; *n* = 4 mice) (Fig. 5*A*,*B*), while colocalization with vGluT1 mRNA was hardly detectable in the scattered BiP-expressing cells adjacent to the BiP/vGluT1 mRNA-enriched pyramidal cell layer (0.4 ± 0.7% of the BiP-positive cells coexpressed vGluT1; *n* = 4 mice, *t*_(6)_ = 18.48, *p* < 0.0001 from BiP^+^GAD65/67^+^ cells, *t* test) (Fig. 5*C*,*D*). In the DG, the distribution of BiP mRNA between disseminated GAD65/67 mRNA-expressing neurons (Fig. 5*A*,*B*) and vGluT1 mRNA-expressing neurons (Fig. 5*C*,*D*) was more balanced, although again with a preference toward inhibitory cells (47.0 ± 9.9% and 30.0 ± 7.2% of the BiP-positive cells coexpressed GAD65/67 or vGluT1, respectively; *n* = 4 mice, *t*_(6)_ = 1.392, *p* = 0.2133, *t* test).

**Figure 6.**
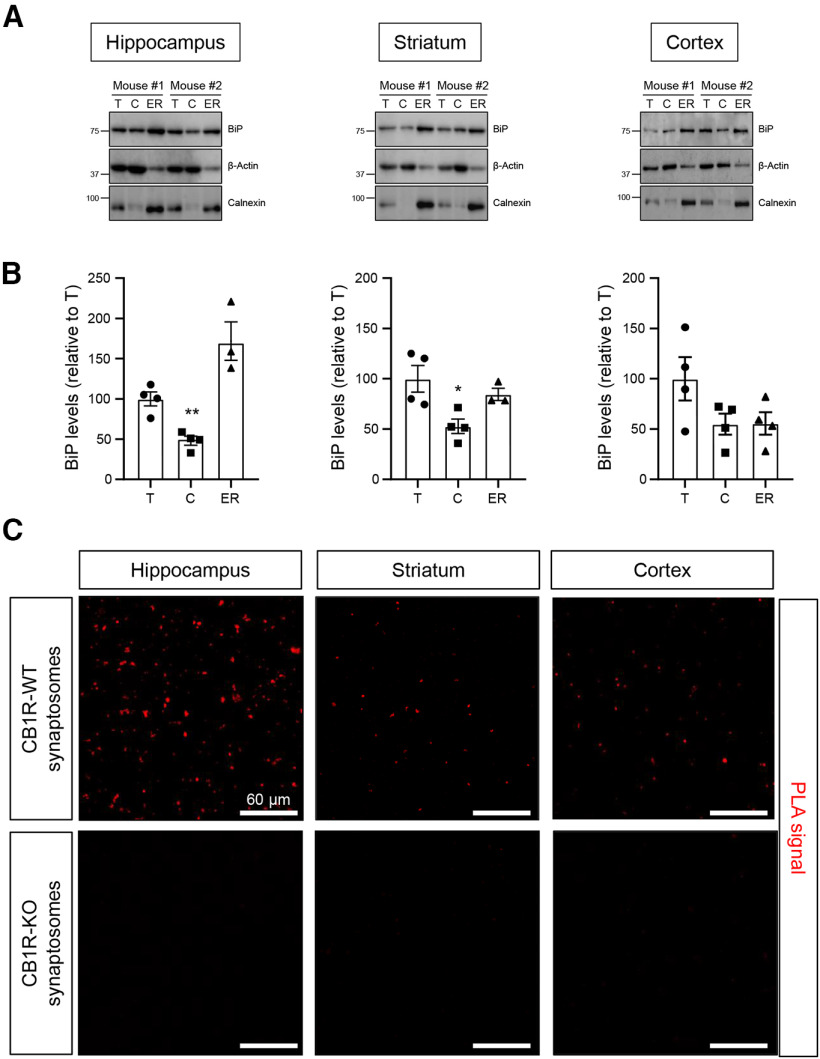
Subcellular localization of BiP in the mouse brain. ***A***, Western blotting of BiP in total-extract (T), cytosolic (C), and ER fractions from the hippocampus, striatum, and cortex of 3- to 4-month-old WT mice. Calnexin was included as an ER-specific marker. Representative blots from 2 mice are shown. ***B***, Quantification of BiP levels in the C and ER fractions relative to BiP levels in the T fraction. *p < 0.05, **p < 0.01 from the corresponding ER fraction by one-way ANOVA with Tukey's multiple comparisons test (*n* = 3 or 4 mice per group). ***C***, PLA experiments conducted on synaptosomal fractions isolated from the hippocampus, striatum, and cortex of 3- to 4-month-old CB1R-WT and CB_1_R-KO mice. Representative images of hippocampal (left column), striatal (middle column), and cortical (right column) synaptosomes, with CB_1_R-BiP complexes depicted in red, are shown (*n* = 5 mice per group).

The most widely reported subcellular localization of BiP is the ER lumen, while CB_1_R is largely located at the plasma membrane, and its CTD faces the cytoplasm since its biosynthesis starts on the ER. To assess this apparent inconsistency, we performed subcellular fractionation experiments in mouse brain samples. Analysis of hippocampal, striatal, and cortical tissue extracts showed that BiP is present not only in the ER but also in the cytosolic fraction (Fig. 6*A*,*B*) [Hippocampus: cytosol (*n* = 4 mice) vs ER (*n* = 3 mice), *F*_(2,8)_ = 21.50, *p* = 0.0004, ANOVA; striatum: cytosol (*n* = 4 mice) vs ER (*n* = 3 mice), *F*_(2,8)_ = 6.232, *p* = 0.0234, ANOVA; cortex: cytosol (*n* = 4 mice) vs ER (*n* = 4 mice), *F*_(2,9)_ = 2.858, *p* = 0.9993, ANOVA]. This observation supports the notion that cytoplasmic BiP binds to CB_1_R-CTD, and aligns with previous reports showing that not all BiP functions can be attributed to its interaction with ER-resident proteins (Belfi et al., 1999; Cha-Molstad et al., 2015; Shim et al., 2018; Yoon et al., 2018), and that a population of BiP molecules is found adjacent to the plasma membrane (Tsai et al., 2015). As the majority of CB_1_R resides at the presynapse, where it controls neurotransmitter release (Piomelli, 2003), we also evaluated whether CB_1_R-BiP complexes are present in this subcellular location. PLA analyses revealed a pronounced positive signal in synaptosomes from the hippocampus, striatum, and cortex of CB1R-WT mice, but not of CB_1_R-KO littermates (Fig. 6*C*).

**Figure 7.**
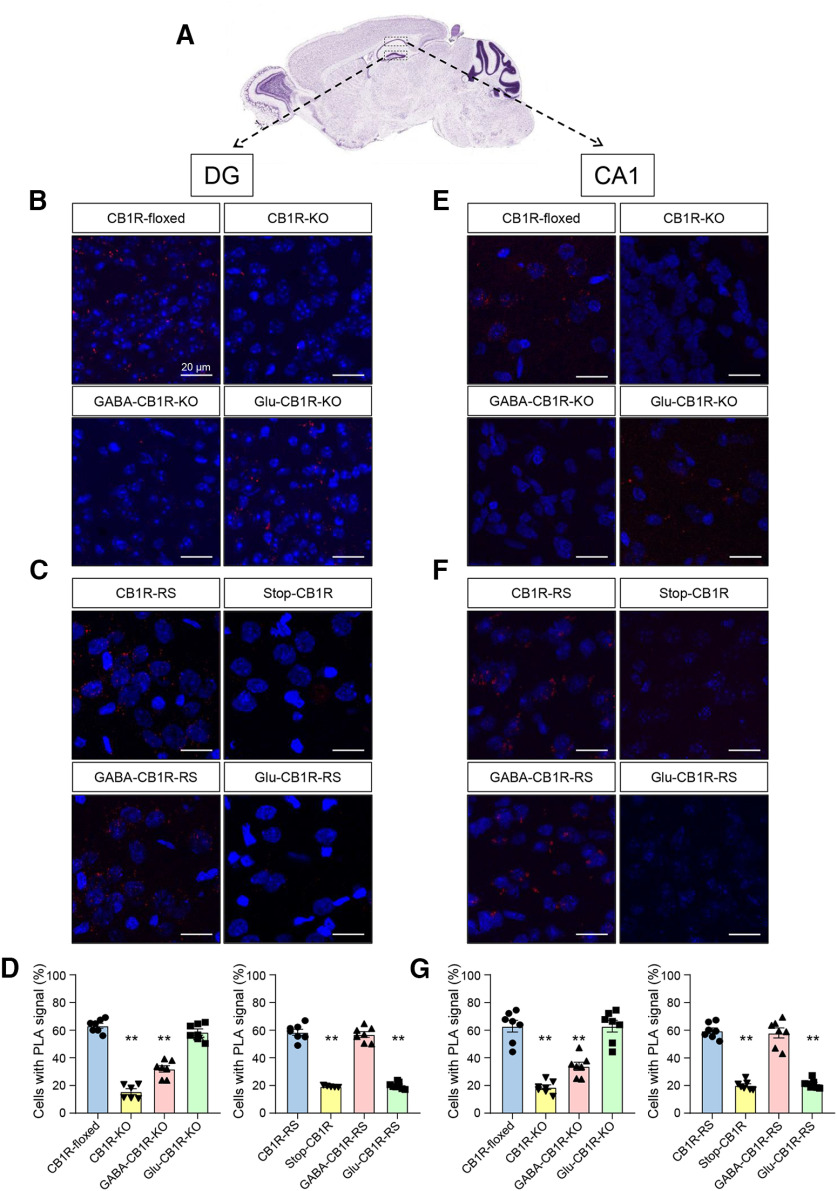
CB_1_R-BiP complexes reside on GABAergic terminals of the mouse hippocampus. ***A***, PLA experiments were conducted on hippocampal sections from 3- to 4-month-old mice of different genotypes. Representative low-magnification image and selected regions for analysis are shown. Image credit: Allen Institute. In the rest of the panels, CB_1_R-BiP complexes are shown as red dots, and nuclei are colored in blue by DAPI staining. ***B***, Representative images of dentate gyrus (DG) sections from CB_1_R-floxed, CB_1_R-KO, GABA-CB_1_R-KO, and Glu-CB_1_R-KO mice. ***C***, Representative images of DG sections from Stop-CB_1_R, CB_1_R-RS, GABA-CB_1_R-RS, and Glu-CB_1_R-RS mice. ***D***, Quantification of the number of cells containing one or more dots expressed as the percentage of the total number of cells (DAPI-stained nuclei) in DG sections. **p < 0.01 from the corresponding CB_1_R-floxed group or the corresponding CB_1_R-RS group by one-way ANOVA with Tukey's multiple comparisons test (*n* = 6 or 7 fields from 3 different animals per group). ***E***, Representative images of CA1 sections from CB_1_R-floxed, CB_1_R-KO, GABA-CB_1_R-KO, and Glu-CB_1_R-KO mice. ***F***, Representative images of CA1 sections from Stop-CB_1_R, CB_1_R-RS, GABA-CB_1_R-RS, and Glu-CB_1_R-RS mice. ***G***, Quantification of the number of cells containing one or more dots expressed as the percentage of the total number of cells (DAPI-stained nuclei) in CA1 sections. **p < 0.01 from the corresponding CB_1_R-floxed group or the corresponding CB_1_R-RS group by one-way ANOVA with Tukey's multiple comparisons test (*n* = 6 or 7 fields from 3 different animals per group).

**Figure 8.**
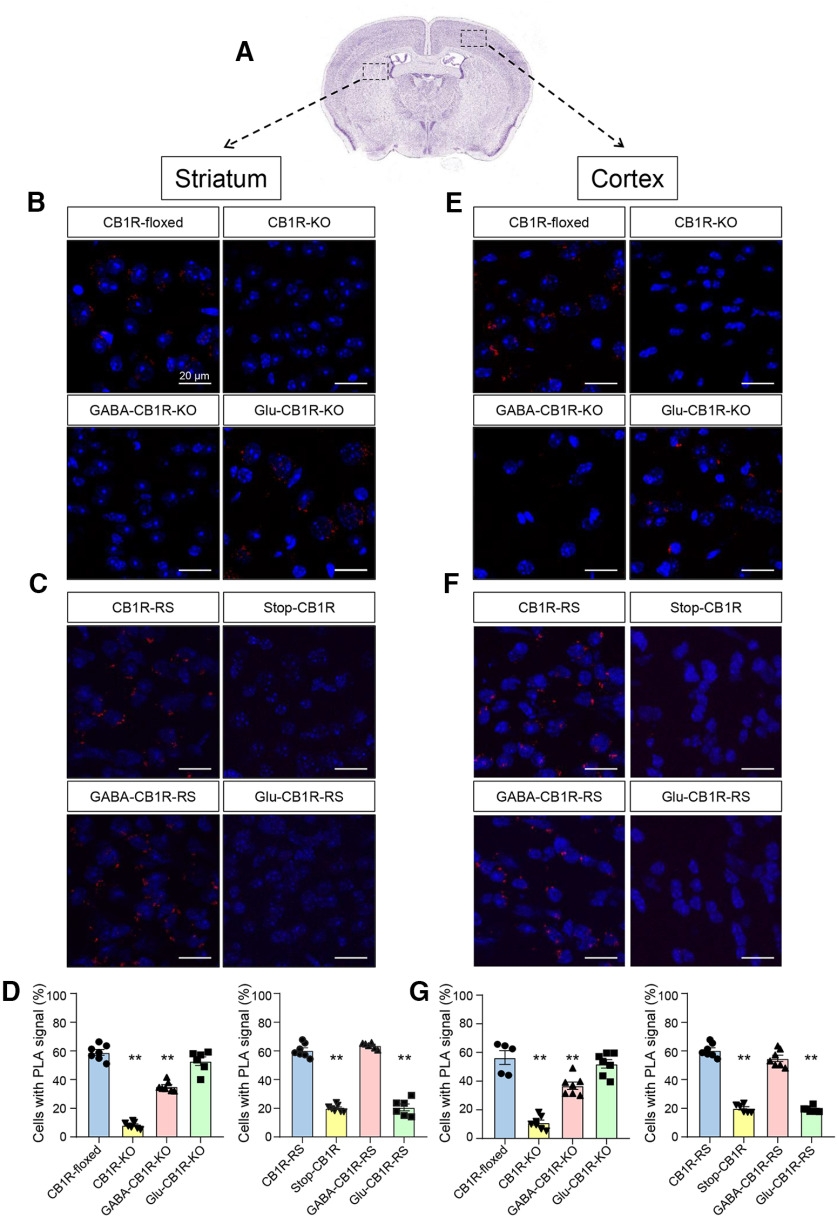
CB_1_R-BiP complexes reside on GABAergic terminals of the mouse striatum and cortex. ***A***, PLA experiments were conducted on striatal and cortical sections from 3- to 4-month-old mice of different genotypes. Representative low-magnification image and selected regions for analysis are shown. Image credit: Allen Institute. In the rest of the panels, CB_1_R-BiP complexes are shown as red dots, and nuclei are colored in blue by DAPI staining. ***B***, Representative images of striatal sections from CB_1_R-floxed, CB_1_R-KO, GABA-CB_1_R-KO, and Glu-CB_1_R-KO mice. ***C***, Representative images of striatal sections from Stop-CB_1_R, CB_1_R-RS, GABA-CB_1_R-RS, and Glu-CB_1_R-RS mice. ***D***, Quantification of the number of cells containing one or more dots expressed as the percentage of the total number of cells (DAPI-stained nuclei) in striatal sections. **p < 0.01 from the corresponding CB_1_R-floxed group or the corresponding CB_1_R-RS group by one-way ANOVA with Tukey's multiple comparisons test (*n* = 6 or 7 fields from 3 different animals per group). ***E***, Representative images of cortical sections from CB_1_R-floxed, CB_1_R-KO, GABA-CB_1_R-KO, and Glu-CB_1_R-KO mice. ***F***, Representative images of cortical sections from Stop-CB_1_R, CB_1_R-RS, GABA-CB_1_R-RS, and Glu-CB_1_R-RS mice. ***G***, Quantification of the number of cells containing one or more dots expressed as the percentage of the total number of cells (DAPI-stained nuclei) in cortical sections. **p < 0.01 from the corresponding CB_1_R-floxed group or the corresponding CB_1_R-RS group by one-way ANOVA with Tukey's multiple comparisons test (*n* = 6 or 7 fields from 3 different animals per group).

Next, to obtain a detailed neuroanatomical map of CB_1_R-BiP protein complexes, we conducted *in situ* PLA assays on brain slices from various genetic mouse models of conditional loss or gain of CB_1_R expression (Fig. 7*A*). We first used hippocampi from conditional CB_1_R-KO models (Marsicano et al., 2002) (Fig. 7*B-G*). PLA experiments conducted on hippocampal sections from control adult CB_1_R*^floxed/floxed^* (hereafter CB_1_R-floxed) mice showed that 63.2 ± 4.7% and 62.9 ± 11.2% of the cells contained positive puncta in the DG and CA1, respectively (*n* = 6 or 7 fields from 3 different mice, *t*_(12)_ = 0.074, *p* = 0.9424, *t* test). This signal was strongly reduced in sections from CB_1_R*^floxed/floxed;CMV-Cre^* (hereafter CB_1_R-KO) mice (DG: 14.8 ± 5.0%; *n* = 6 fields from 3 different mice, *F*_(3,23)_ = 109.6, *p* < 0.0001 from CB_1_R-floxed mice, ANOVA. CA1: 18.8 ± 4.5%; *n* = 7 fields from 3 different mice, *F*_(3,24)_ = 40.86, *p* < 0.0001 from CB_1_R-floxed mice, ANOVA). In conditional KO mice in which the gene encoding CB_1_R had been selectively deleted from forebrain GABAergic neurons (CB_1_R*^floxed/floxed;Dlx5/6-Cre^*; hereafter GABA- CB_1_R-KO), we found a notable decrease in the percentage of cells expressing positive dots (DG: 31.9 ± 6.2%; *n* = 7 fields from 3 different mice, *F*_(3,23)_ = 109.6, *p* < 0.0001 from CB_1_R-floxed mice, ANOVA. CA1: 33.9 ± 7.8%, *n* = 7 fields from 3 different mice, *F*_(3,24)_ = 40.86, *p* < 0.0001 from CB_1_R-floxed mice, ANOVA). In contrast, sections from mice in which the gene encoding CB_1_R had been selectively deleted from dorsal telencephalic glutamatergic neurons (CB_1_R*^floxed/floxed;Nex1-Cre^*; hereafter Glu-CB_1_R-KO) displayed a similar pattern of PLA staining than their CB_1_R-floxed counterparts (DG: 58.6 ± 5.9%; *n* = 7 fields from 3 different mice, *F*_(3,23)_ = 109.6, *p* = 0.3052 from CB_1_R-floxed mice, ANOVA. CA1: 60.8 ± 1.1%; *n* = 7 fields from 3 different mice, *F*_(3,24)_ = 40.86, *p* > 0.9999 from CB_1_R-floxed mice, ANOVA). Comparable overall data were obtained in sections from mouse striatum (Fig. 8*B*,*D*) [CB_1_R-KO (*n* = 7 fields from 3 different mice) vs CB_1_R-floxed (*n* = 7 fields from 3 different mice): *F*_(3,23)_ =151.4, *p* < 0.0001, ANOVA; GABA-CB_1_R-KO (*n* = 7 fields from 3 different mice) vs CB_1_R-floxed (*n* = 7 fields from 3 different mice): *F*_(3,23)_ = 151.4, *p* < 0.0001, ANOVA; Glu-CB_1_R-KO (*n* = 6 fields from 3 different mice) vs CB_1_R-floxed (*n* = 7 fields from 3 different mice): *F*_(3,23)_ = 151.4, *p* = 0.0850, ANOVA] and cortex (Fig. 8*E*,*G*) [CB_1_R-KO (*n* = 7 fields from 3 different mice) vs CB_1_R-floxed (*n* = 6 fields from 3 different mice): *F*_(3,22)_ = 48.30, *p* < 0.0001, ANOVA; GABA-CB_1_R-KO (*n* = 7 fields from 3 different mice) vs CB_1_R-floxed (*n* = 6 fields from 3 different mice): *F*_(3,22)_ = 48.30, *p* = 0.0006, ANOVA; Glu-CB_1_R-KO (*n* = 7 fields from 3 different mice) vs CB_1_R-floxed (*n* = 6 fields from 3 different mice): *F*_(3,22)_ = 48.30, *p* = 0.6079, ANOVA].

We subsequently made use of a Cre-mediated, lineage-specific, CB_1_R gene expression-rescue strategy from a CB1R-null background (hereafter Stop-CB_1_R mice) (De Salas-Quiroga et al., 2015; De Giacomo et al., 2020a) (Fig. 7*C-G*). PLA assays in hippocampal sections from these mice showed, as expected, a marginal CB_1_R-KO-like background signal (DG: 20.1 ± 3.2%, CA1: 21.2 ± 3.2%; *n* = 5 or 7 fields from 3 different mice, respectively; *t*_(10)_ = 0.5326, *p* = 0.6060, *t* test). In line with the data from conditional KO mice, rescuing CB_1_R gene expression in Stop-CB_1_R mice with a constitutive Cre recombinase (Stop-CB_1_R*^EIIa-Cre^*, hereafter, CB_1_R-RS) restored CB_1_R-BiP complexes to the levels of control CB_1_R-floxed mice (DG: 59.6 ± 5.5%, CA1: 58.5 ± 5.8%; *n* = 5 or 7 fields from 3 different mice, respectively; *F*_(3,24)_ = 94.99, *p* < 0.0001, ANOVA; and *F*_(3,22)_ = 121.6, *p* < 0.0001, ANOVA, respectively). This effect was paralleled in brain sections from conditionally rescued Stop-CB_1_R*^Dlx5/6-Cre^* (hereafter, GABA-CB_1_R-RS) mice [DG: 58.1 ± 9.6%; *n* = 7 fields from 3 different mice; *F*_(3,24)_ = 94.99, *p* = 0.9279 from CB_1_R-RS mice, ANOVA. CA1: 56.9 ± 5.5%; *n* = 7 fields from 3 different mice; *F*_(3,22)_ = 121.6, *p* = 0.8400 from CB_1_R-RS mice, ANOVA], but not from conditionally rescued Stop-CB_1_R*^Nex1-Cre^* [hereafter, Glu-CB_1_R-RS mice (DG: 21.1 ± 3.2%; *n* = 7 fields from 3 different mice; *F*_(3,24)_ = 94.99, *p* < 0.0001 from CB_1_R-RS mice, ANOVA. CA1: 20.0 ± 2.5%; *n* = 7 fields from 3 different mice; *F*_(3,22)_ = 121.6, *p* < 0.0001 from CB_1_R-RS mice, ANOVA]. As in the aforementioned conditional KO mouse experiments, these CB_1_R gene expression-rescue data in the mouse hippocampus displayed a similar global pattern in the mouse striatum (Fig. 8*C*,*D*) [Stop-CB_1_R (*n* = 7 fields from 3 different mice) vs CB_1_R-RS (*n* = 7 fields from 3 different mice): *F*_(3,22)_ = 230.5, *p* < 0.0001, ANOVA; GABA-CB_1_R-RS (*n* = 6 fields from 3 different mice) vs CB_1_R-RS (*n* = 7 fields from 3 different mice): *F*_(3,22)_ = 230.5, *p* = 0.3465, ANOVA; Glu-CB_1_R-RS (*n* = 6 fields from 3 different mice) vs CB_1_R-RS (*n* = 7 fields from 3 different mice): *F*_(3,22)_ = 230.5, *p* < 0.0001, ANOVA] and cortex (Fig. 8*F*,*G*) [Stop-CB_1_R (*n* = 6 fields from 3 different mice) vs CB_1_R-RS (*n* = 7 fields from 3 different mice): *F*_(3,22)_ = 167.0, *p* < 0.0001, ANOVA; GABA-CB_1_R-RS (*n* = 7 fields from 3 different mice) vs CB_1_R-RS (*n* = 7 fields from 3 different mice): *F*_(3,22)_ = 167.0, *p* = 0.0620, ANOVA; Glu-CB_1_R-RS (*n* = 6 fields from 3 different mice) vs CB_1_R-RS (*n* = 7 fields from 3 different mice): *F*_(3,22)_ = 167.0, *p* < 0.0001, ANOVA].

Together, these data support the interaction between CB_1_R and BiP in three key regions of the mouse brain, and, more specifically, a restricted occurrence of CB_1_R-BiP complexes in GABAergic neurons.

### BiP affects CB_1_R function *in vivo*

**Figure 9.**
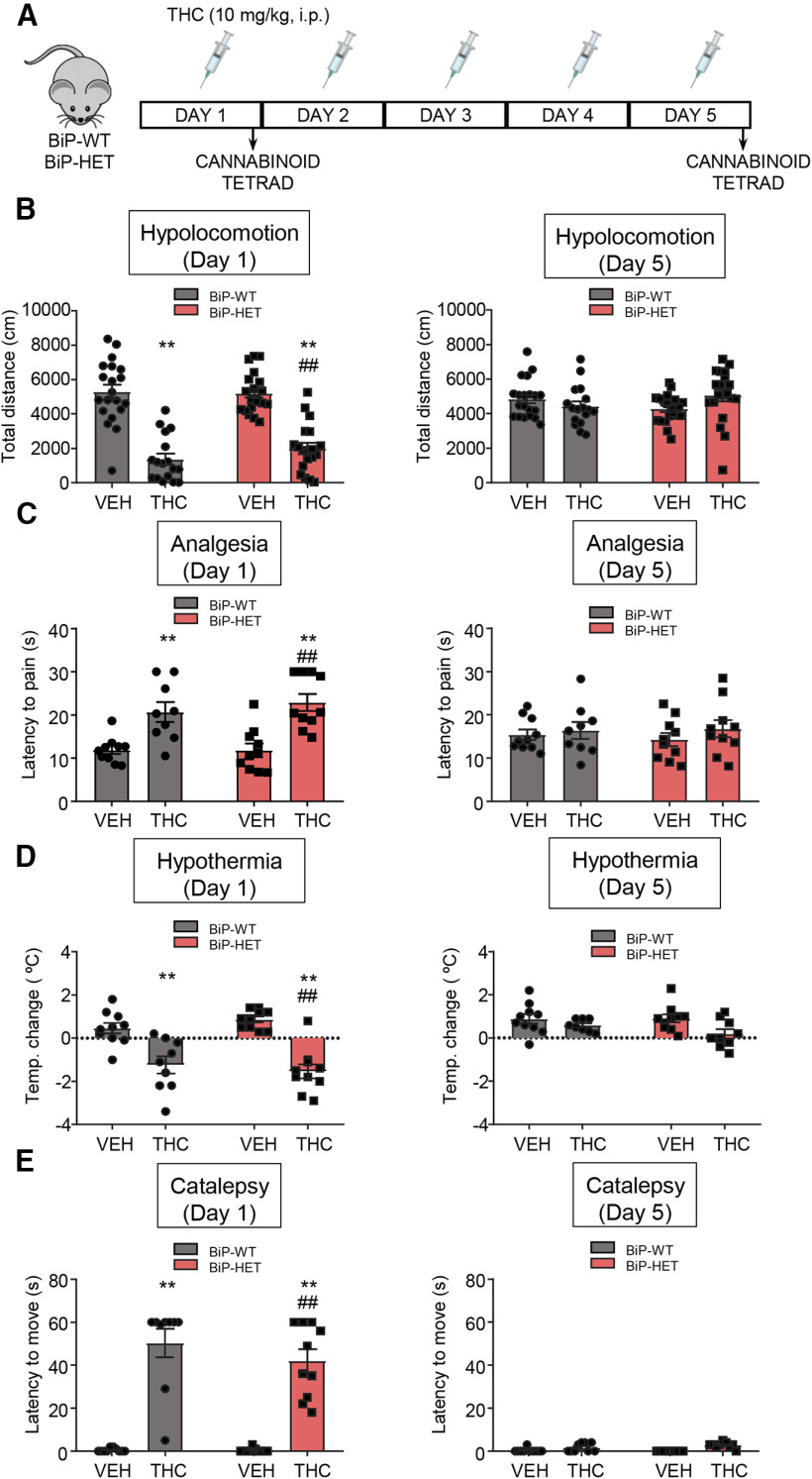
BiP does not affect CB_1_R-evoked hypolocomotion, analgesia, hypothermia, and catalepsy *in vivo*. ***A***, Scheme of the experiments. Vehicle or THC (10 mg/kg, 1 i.p. injection per day) was administered for 5 d to 3- to 4-month-old BiP^+/+^ (BiP-WT) and BiP^+/−^ (BiP-HET) mice. The “cannabinoid tetrad” was evaluated on days 1 and 5, starting 30 min after the corresponding acute-drug injections. ***B***, Ambulation (total distance traveled, cm) in the open-field test on day 1 (left) and day 5 (right). ***C***, Analgesia (latency to pain, s) in the hotplate test on day 1 (left) and day 5 (right). ***D***, Hypothermia (change in body temperature, °C) as measured with a rectal thermometer on day 1 (left) and day 5 (right). ***E***, Catalepsy (latency to move, s) as measured on a horizontal bar on day 1 (left) and day 5 (right). ***B-E***: **p < 0.01 from the corresponding vehicle group; ^##^*p* < 0.01 from the BiP-WT-vehicle group; two-way ANOVA with Tukey's multiple comparisons test (***B***, *n* = 17-20 mice per group; ***C-E***, *n* = 9 or 10 mice per group).

THC induces numerous behavioral changes in laboratory animals and humans. The combination of hypolocomotion, analgesia, catalepsy, and hypothermia, usually designated as the “cannabinoid tetrad,” has evolved as a powerful tool to identify pharmacological or genetic interventions that target CB_1_R (Martin, 1986; Metna-Laurent et al., 2017). Previous studies have shown that these four behavioral traits rely selectively on the activation of CB_1_R molecules located on various populations of glutamatergic or dopamine D_1_ receptor-expressing projection neurons, but not on GABAergic interneurons, thus allowing a neurobiological correlate between CB_1_R cellular expression and function (Monory et al., 2007; De Giacomo et al., 2020a). We studied the “cannabinoid tetrad” in BiP-HET and BiP-WT littermates (Fig. 9*A*), and found that acute THC injection (10 mg/kg, i.p.) elicited the four archetypical effects of the “cannabinoid tetrad” to the same extent in BiP-HET and BiP-WT animals (Fig. 9, left panels) [Hypolocomotion: BiP-WT-vehicle (*n* = 19 mice) vs BiP-WT-THC (*n* = 17 mice), *F*_(1,72)_ = 111.9, *p* < 0.0001, ANOVA; BiP-HET-vehicle (*n* = 20 mice) vs BiP-HET-THC (*n* = 19 mice), *F*_(1,72)_ = 111.9, *p* < 0.0001, ANOVA. Analgesia: BiP-WT-vehicle (*n* = 10 mice) vs BiP-WT-THC (*n* = 9 mice), *F*_(1,35)_ = 32.93, *p* = 0.0059, ANOVA; BiP-HET-vehicle (*n* = 10 mice) vs BiP-HET-THC (*n* = 10 mice), *F*_(1,35)_ = 32.93, *p* = 0.0030, ANOVA. Hypothermia: BiP-WT-vehicle (*n* = 9 mice) vs BiP-WT-THC (*n* = 9 mice), *F*_(1,35)_ = 50.76, *p* = 0.0012, ANOVA; BiP-HET-vehicle (*n* = 9 mice) vs BiP-HET-THC (*n* = 8 mice), *F*_(1,35)_ = 50.76, *p* = <0.0001, ANOVA. Catalepsy: BiP-WT-vehicle (*n* = 10 mice) vs BiP-WT-THC (*n* = 9 mice), *F*_(1,35)_ = 124.5, *p* < 0.0001, ANOVA; BiP-HET-vehicle (*n* = 9 mice) vs BiP-HET-THC (*n* = 9 mice), *F*_(1,35)_ = 124.5, *p* < 0.0001, ANOVA]. In addition, following a 5 d sustained treatment, BiP-HET and BiP-WT mice developed a comparable tolerance to THC (Fig. 9, right panels) [Hypolocomotion: BiP-WT-vehicle (*n* = 20 mice) vs BiP-WT-THC (*n* = 17 mice), *F*_(1,73)_ = 0.4632, *p* = 0.7219, ANOVA; BiP-HET-vehicle (*n* = 20 mice) vs BiP-HET-THC (*n* = 20 mice), *F*_(1,73)_ =0.4632, *p* = 0.1704, ANOVA. Analgesia: BiP-WT-vehicle (*n* = 10 mice) vs BiP-WT-THC (*n* = 9 mice), *F*_(1,35)_ = 1.094, *p* = 0.9759, ANOVA; BiP-HET-vehicle (*n* = 10 mice) vs BiP-HET-THC (*n* = 10 mice), *F*_(1,35)_ = 1.094, *p* = 0.7068, ANOVA. Hypothermia: BiP-WT-vehicle (*n* = 10 mice) vs BiP-WT-THC (*n* = 8 mice), *F*_(1,33)_ = 6.741, *p* = 0.7040, ANOVA; BiP-HET-vehicle (*n* = 10 mice) vs BiP-HET-THC (*n* = 9 mice), *F*_(1,33)_ = 6.741, *p* = 0.0609, ANOVA. Catalepsy: BiP-WT-vehicle (*n* = 10 mice) vs BiP-WT-THC (*n* = 9 mice), *F*_(1,35)_ = 7.437, *p* = 0.2262, ANOVA; BiP-HET-vehicle (*n* = 10 mice) vs BiP-HET-THC (*n* = 10 mice), *F*_(1,35)_ = 7.437, *p* = 0.2440, ANOVA].

**Figure 10.**
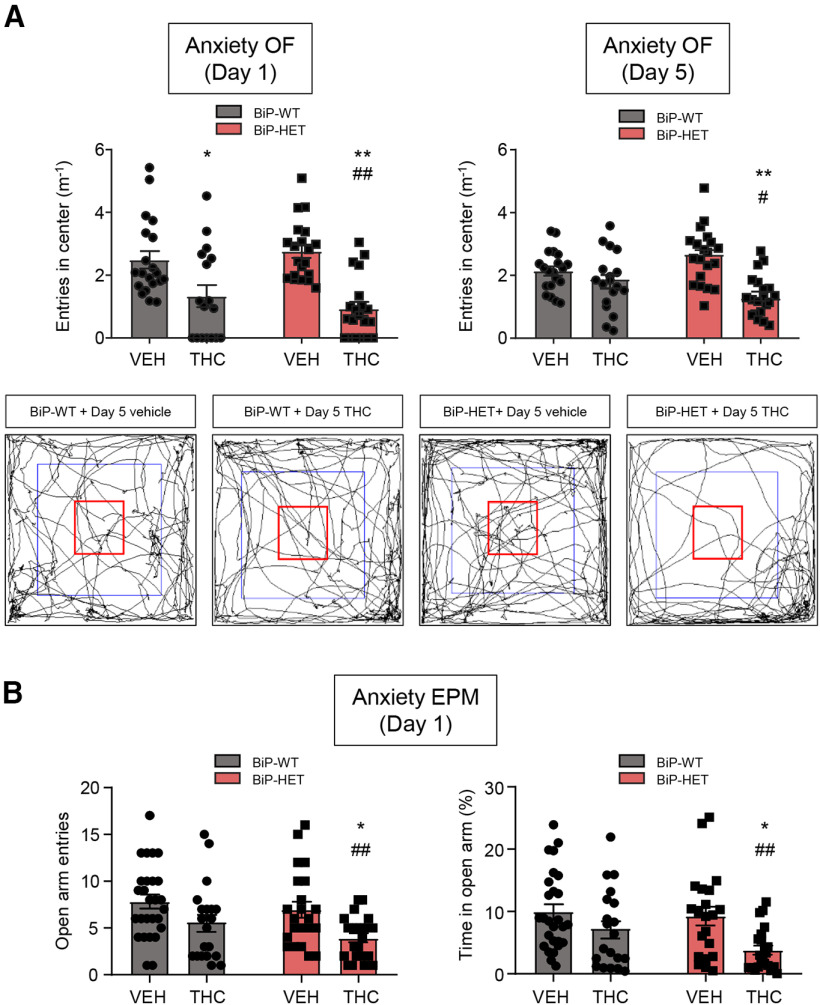
BiP modulates CB_1_R-evoked anxiety *in vivo*. Anxiety-like behaviors were measured on an experimental scheme similar to that shown in Figure 9*A*. ***A***, Anxiety (normalized entries in the center, m^−1^) in the open-field (OF) test on day 1 (left) and day 5 (right). Arenas (with their centers outlined in red) illustrating the ambulation of a representative animal per group on day 5 are shown (bottom). ***B***, Anxiety (left: number of entries in the open arms; right: time spent in the open arms, %) in the elevated plus maze (EPM) test on day 1. ***A***, ***B***: *p < 0.05, **p < 0.01 from the corresponding vehicle group, or ^#^*p* < 0.05, ^##^*p* < 0.01 from the BiP-WT-vehicle group; two-way ANOVA with Tukey's multiple comparisons test (***A***, *n* = 18-20 mice per group; ***B***, *n* = 20-27 mice per group).

As the CB_1_R-BiP complexes reside selectively on GABAergic neurons (see above), it is not surprising that the deletion of a BiP allele does not modify any of the classical “cannabinoid tetrad” behavioral traits. Of note, anxiety-like behaviors induced by cannabinoid intoxication have been shown to rely selectively on the activation of CB_1_R molecules located on GABAergic interneurons (Rey et al., 2012; De Giacomo et al., 2020a,b). Because the open-field test of the “cannabinoid tetrad” can also be used to define anxious phenotypes by evaluating the relative ambulation of the animals across the center of the arena (Seibenhener and Wooten, 2015), we conducted these analyses in our experimental setting. A single THC injection reduced the ambulation of the mice across the center of the arena equally in BiP-HET and BiP-WT mice (Fig. 10*A*, left) [BiP-WT-vehicle (*n* = 20 mice) vs BiP-WT-THC (*n* = 17 mice): *F*_(1,73)_ = 32.35, *p* = 0.0164, ANOVA; BiP-HET-vehicle (*n* = 20 mice) vs BiP-HET-THC (*n* = 20 mice): *F*_(1,73)_ = 32.35, *p* < 0.0001, ANOVA]. However, after a 5 d continuing THC treatment, the ambulation across the center of the arena remained lowered by acute THC in BiP-HET mice but not in their BiP-WT littermates (Fig. 10*A*, right) [BiP-WT-vehicle (*n* = 20 mice) vs BiP-WT-THC (*n* = 18 mice): *F*_(1,74)_ = 20.54, *p* = 0.5226, ANOVA; BiP-HET-vehicle (*n* = 20 mice) vs BiP-HET-THC (*n* = 20 mice): *F*_(1,74)_ = 20.54, *p* < 0.0001, ANOVA].

To provide further support to the control of CB_1_R-mediated anxiety by BiP, we used the elevated plus maze test, a widely recognized measure of anxiety that served originally to define the anxiogenic activity of the CB_1_R pool located on GABAergic neurons (Rey et al., 2012). We injected BiP-WT and BiP-HET mice with vehicle or THC (10 mg/kg, i.p.), and found that the drug induced only an anxiogenic trend in BiP-WT mice but a significant anxiogenic effect in BiP-HET littermates, as evidenced by the decrease in both the number of entries (Fig. 10*B*, left) [BiP-WT-vehicle (*n* = 27 mice) vs BiP-WT-THC (*n* = 20 mice): *F*_(1,86)_ = 11.51, *p* = 0.1471, ANOVA; BiP-HET-vehicle (*n* = 21 mice) vs BiP-HET-THC (*n* = 22 mice): *F*_(1,86)_ = 11.51, *p* = 0.0470, ANOVA] and the time of permanence (Fig. 10*B*, right) in the open arms of the device [BiP-WT-vehicle (*n* = 27 mice) vs BiP-WT-THC (*n* = 20 mice): *F*_(1,86)_ = 11.34, *p* = 0.3385, ANOVA; BiP-HET-vehicle (*n* = 21 mice) vs BiP-HET-THC (*n* = 22 mice): *F*_(1,86)_ = 11.34, *p* = 0.0155, ANOVA].

Together, these data support that BiP, by interacting with CB_1_R on GABAergic neurons, modulates anxiety-like behaviors on cannabinoid administration.

## Discussion

Here, we show that BiP interacts specifically with CB_1_R-CTD. BiP is known to interact with some GPCRs during their folding (Siffroi-Fernandez et al., 2002; Mizrachi and Segaloff, 2004; Langer et al., 2008), and has been found associated to melanocortin MC_4_ receptors at the plasma membrane (Yoon et al., 2018). The CB_1_R-BiP interaction occurs between a short amino-acid stretch in the CB_1_R-CTD and the BiP-SBDα domain. The latter domain, to our knowledge, has never been implicated in the binding of BiP to membrane receptors. As the protein-binding/refolding function of BiP is usually ascribed to its SBDβ domain (Yang et al., 2015, 2017), we cannot rule out that additional proteins interact through this region onto the CB_1_R-BiP complexes. The BiP-interacting region in CB_1_R partially overlaps with the putative *C*-terminal helix 9 of the receptor (Ahn et al., 2009), which might serve as an axon-targeting signal and a potential protein–protein interaction site (Fletcher-Jones et al., 2019). How the synaptic trafficking of CB_1_R could be controlled by BiP is therefore an intriguing possibility that remains to be explored. Additionally, the BiP-binding region of CB_1_R contains a specific phosphorylation site (S452) that regulates this protein–protein interaction, and may conceivably participate in agonist-induced receptor signaling and subsequent internalization (Daigle et al., 2008). Indeed, a high-throughput phosphoproteomic study identified this phosphorylated residue in the mouse brain (Wiśniewski et al., 2010). The lack of reported mutations in this BiP-binding region of CB_1_R (https://gpcrdb.org), along with its evolutionary conservation, further supports its biological importance.

CB_1_R-evoked signaling is markedly affected on BiP binding. This finding contrasts with the subtle effect of CRIP1a on CB_1_R/G-protein coupling (Blume et al., 2015), and with the BiP-mediated facilitation of melanocortin MC_4_ receptor activation (Yoon et al., 2018). Accruing evidence has linked ERK and Akt/mTORC1 activation to various key CB_1_R-evoked effects in the brain (Rubino et al., 2007; Guegan et al., 2013; Puighermanal et al., 2013; Blázquez et al., 2020). However, the possible relevance of Gα_q/11_ protein in CB_1_R neurobiological action remains unclear (Diez-Alarcia et al., 2016). Our data unveil an unprecedented functional coupling of CB_1_R signaling to Gα_q/11_, as well as a selective hampering effect of BiP on it. Interestingly, regions analogous to CB_1_R helix 9, which overlaps with the BiP-binding site, have been reported to act as Gα_q/11_-binding sites in rhodopsin (Murakami and Kouyama, 2008) and bradykinin B_2_ receptor (Piserchio et al., 2005). Thus, it is conceivable that in CB_1_R the binding of BiP constitutes a competitive steric impediment to achieve Gα_q/11_ binding and activation.

Our detailed mapping of CB_1_R-BiP complexes in the mouse brain shows that GABAergic neurons constitute the foremost cell population expressing these complexes. This is in line with a previous high-throughput proteomic study showing that BiP coimmunoprecipitates with CB_1_R in mouse GABAergic neurons (Mattheus et al., 2016). It is worth noting that, despite their low CB_1_R/G-protein coupling efficacy (Steindel et al., 2013), GABAergic terminals contain large amounts of CB_1_R (Marsicano and Lutz, 1999; Katona and Freund, 2008) likely displaying a high tonic activity (Roberto et al., 2010). Given its inhibitory role, we speculate that BiP binding may represent a counterpoint to ensure a balanced CB_1_R activity in the physiological control of glutamatergic/GABAergic neurotransmission. More specifically, THC-elicited anxiety relies on mTORC1 activation on engagement of CB_1_R on hippocampal GABAergic interneurons (Rey et al., 2012; Puighermanal et al., 2013; De Giacomo et al., 2020a,b). In addition, a role of Gα_q/11_ protein-coupled receptors (e.g., serotonin 5-HT_2C_ receptor) in the induction of anxiety has been suggested (Mazzone et al., 2018). Thus, we propose that the THC-evoked high-input activation of a restricted Gα_q/11_-coupled pool of CB_1_R molecules located on hippocampal GABAergic interneurons, via the mTORC1 signaling axis, triggers anxiety-like behaviors, a process plausibly controlled by BiP binding to CB_1_R at the presynapse. This would provide an unprecedented mechanism for the spatially selective control of CB_1_R signaling in the brain, and supports that favoring CB_1_R-BiP association would reduce anxiety, a frequent negative effect of CB_1_R overactivation. As CB_1_R-BiP complexes also reside on GABAergic neurons in other brain regions as the cortex and striatum, the possibility that BiP binding controls additional CB1R-related behaviors remains to be determined.
